# Hemoporfin Photodynamic Therapy for Port-Wine Stain: A Randomized Controlled Trial

**DOI:** 10.1371/journal.pone.0156219

**Published:** 2016-05-26

**Authors:** Yi Zhao, Ping Tu, Guoyu Zhou, Zhanchao Zhou, Xiaoxi Lin, Huilan Yang, Zhong Lu, Tianwen Gao, Yating Tu, Hongfu Xie, Qingshan Zheng, Ying Gu, Jining Tao, Xuejun Zhu

**Affiliations:** 1 Department of Dermatology and Venereology, Peking University First Hospital, Beijing, China; 2 Department of Oral Maxillofacial Surgery, Ninth People’s Hospital Affiliated to Shanghai Jiao Tong University School of Medicine. Shanghai, China; 3 Institute of Dermatology, Chinese Academy of Medical Sciences and Peking Union Medical College, Nanjing, China; 4 Department of Plastic and Reconstructive Surgery, Ninth People’s Hospital Affiliated to Shanghai Jiao Tong University School of Medicine. Shanghai, China; 5 Department of Dermatology, The General Hospital of Guangzhou Military Command, Guangzhou, China; 6 Department of Dermatology, Huashan Hospital, Fudan University, Shanghai, China; 7 Institute of Dermatology of Chinese PLA, Xijing Hospital, Fourth Military Medical University, Xi’an, China; 8 Department of Dermatology, Union Hospital of Tongji Medical College of Huazhong University of Science and Technology, Wuhan, China; 9 Department of Dermatology, Xiangya Hospital Central South University, Changsha, China; 10 Center for Drug Clinical Research, Shanghai University of Chinese Traditional Medicine, Shanghai, China; 11 Department of Laser Medicine, Chinese PLA General Hospital, Beijing, China; 12 Shanghai Fudan-Zhangjiang Bio-Pharmaceutical Co., Ltd., Shanghai, China; The Lee Kong Chian School of Medicine, SINGAPORE

## Abstract

**Background and Objectives:**

Photodynamic therapy (PDT) has shown potentially beneficial results in treating port-wine stain, but its benefit–risk profile remains undefined. This study aimed to evaluate the efficacy and safety of PDT conducted with hemoporfin and a 532 nm continuous wave laser to treat port-wine stain clinically.

**Patients and Methods:**

This randomized clinical trial was conducted in eight hospitals in China. Participants were adolescent and adult patients (age range: 14–65 years old) with port-wine stain. During stage 1 (day 1 to week 8) all patients were randomized at a 3:1 ratio to treatment (532 nm laser irradiation (96–120 J/cm^2^) with hemoporfin (5mg/kg; PDT-hemoporfin, *n* = 330)) or placebo groups (irradiation with placebo (PDT-placebo, *n* = 110)); during stage 2 (week 8 to 16) patients in both groups were offered treatment. Clinician-evaluators, who were blind to the study, classified each case on the following four-level scale according to assessment of before and after standardized pictures of the lesion area: no improvement: <20%; some improvement: 20–59%; great improvement: 60–89%; or nearly completely resolved: ≥90%. The primary efficacy endpoint was proportion of patients achieving at least some improvement at week 8. The secondary efficacy endpoints were proportion of patients achieving nearly completely resolved or at least great improvement at week 8, proportion of patients achieving early completely resolved, at least great improvement, or at least some improvement at week 16, and the corresponding satisfaction of the investigators and the patients (designated as ‘excellent’, ‘good’, ‘moderate’, or ‘ineffective’) at weeks 8 and 16.

**Results:**

Compared to the PDT-placebo group, the PDT-hemoporfin group showed a significantly higher proportion of patients that achieved at least some improvement (89.7% [*n* = 295; 95% CI, 85.9%-92.5%] vs. 24.5% [*n* = 27; 95% CI, 17.4%-33.3%]) at week 8 (P < 0.0001) and higher improvements for all secondary efficacy endpoints. Treatment reactions occurred in 99.5% (*n* = 731; 95% CI, 98.7%-99.8%) of the PDT-hemoporfin treatments (*n =* 735). Hyperpigmentation occurred in 22.9 per 100 patient-treatments (*n* = 168; 95% CI, 20.0–26.0) in the PDT-hemoporfin treated patients.

**Conclusions:**

Hemoporfin-mediated PDT is an effective and safe treatment option for adolescent and adult patients with port-wine stain.

**Trial Registration:**

Chinese Clinical Trial Registry ChiCTR-TRC-08000213

## Introduction

Port-wine stain (PWS) is the most common congenital vascular malformation reported in 0.3% of infants born worldwide [1]. The visible manifestation of this disorder is often considered a disfigurement and the accompanying social stigma often causes psychological problems for the affected individuals [2]. While no cure for PWS has yet been found, many treatment options have been developed and put into clinical practice; these approaches range from moderately risky (such as covering the PWS with tattoos) to substantially risky (such as radiation), yet cosmetically acceptable results are rarely achieved [3,4]. Even with the current preferred clinical treatment of pulsed-dye laser (PDL), 19–27% of patients achieved ≥75% clearance in randomized clinical trials [5,6], and recurrence or redarkening of the treated PWS occur frequently [7,8]. Therefore, the need for effective and safe modalities to treat PWS remains unfulfilled.

One potential treatment modality is photodynamic therapy (PDT), which uses photosensitizer, light, and oxygen to induce a photochemical reaction that generates highly-reactive singlet oxygen molecules, which are able to cause cell death via apoptosis, necrosis or autophagy [9]. PDT has already been shown to be a successful management tool for treating neoplastic and non-malignant diseases [9,10]. Studies of PDT as a vascular-targeted approach to treat PWS have provided potentially beneficial results [11,12].

PDT using the porphyrin-related photosensitizer hematoporphyrin monomethyl ether (HMME), combined with application of alternative light sources (such as copper vapour laser)**,** appears to represent an effective approach for treating PWS [13,14]. Preliminary studies for PDT using a new product of HMME, hemoporfin, have identified the optimal wavelength as 532 nm [15]. However, the effectiveness and safety of this comprehensive modality (PDT + hemoporfin + 532 nm wavelength) remains to be established by a prospective study of a large population. Therefore, this study was designed as a randomized, double-blind, placebo-controlled phase 3 clinical trial to test the hypothesis that treatment with PDT using an optimized protocol (5mg/kg hemoporfin and 532 nm continuous wave lasers with fluence of 96–120 J/cm^2^) would be effective and safe for patients with PWS.

## Materials and Methods

### Patients

Adolescent and adult patients (age range: 14 to 65 years-old) with clinical diagnosis of PWS were recruited to the study from eight research centers in China (one each located in Beijing, Nanjing, Guangzhou, Xian, Wuhan, and Changsha, and two in Shanghai), all of which are affiliated with large general teaching hospitals. For study enrollment, each patient was required to have adequate renal (serum creatinine and blood urea nitrogen ≤1.5 upper limit of normal [ULN]) and hepatic (alanine aminotransferase and aspartate transaminase ≤1ULN, and total bilirubin ≤1.5 ULN) functions and no history of treatment with isotope, laser or PDT, or systemic treatment for PWS during the past 4 weeks, or topical treatment during the past 2 weeks. Patients were considered ineligible if any one or more of the following conditions were present: other vascular malformations, vessel-related syndromes, or other conditions that might interfere with the study; allergy to porphyrins and analogues; photosensitivity; porphyria; allergic constitution; scar diathesis; immunocompromised conditions; electrocardiographic abnormalities or organic heart diseases; coagulation disorders; psychiatric diseases; severe endocrinopathies; current or one-month previous history of medications that might cause photosensitivity; women who were currently pregnant or lactating.

The study protocols and amendments were approved by the Ethics Committee of Peking University First Hospital. Written informed consent was obtained from all study participants prior to enrollment; for patients younger than 18-years-old, the informed consent was provided by a parent or legal guardian. This study was registered in the Chinese Clinical Trial Registry (Registration number: ChiCTR-TRC-08000213, URL: http://www.chictr.org.cn/showprojen.aspx?proj=9313).

### Interventions

The injectable formulation of hemoporfin (sterile, lyophilized powder) was manufactured in accordance with the national Good Manufacturing Processes (GMP) standard of China, and supplied by Shanghai Fudan-Zhangjiang Bio-Pharmaceutical Co., Ltd. (China). At each center involved in this study, a dedicated manager was assigned for the hemoporfin storage and dispensing.

Before treatment, a treatment-target epidermal site (≤7 cm diameter) was chosen in the PWS area, and the surrounding skin was carefully covered. A fresh working solution of hemoporfin (5 mg/kg, the dose was determined based on our previous studies [16,17]) was prepared by dissolving in normal saline for immediate transfusion (constant speed over 20 min). A group of patients were transfused with normal saline alone and served as the placebo control group. The infusion apparatus was prepared by a nurse and completely covered to avoid potential photodecomposition. At 10 minutes after the transfusion had been initiated, the 532 nm continuous wave laser (see S1 Table for features) was applied to the target site with a power density of 80–100 mW/cm^2^ for a total of 20 minutes; these parameters were used according to pharmacokinetic parameters and the results of a phase IIa study of hemoporfin [16]. Therefore, the fluence was 96–120 J/cm^2^. No allergy testing against hemoporfin was performed prior to the treatment. No anesthesia was required before or after the treatment. Patients were not sedated and wore protective eye goggles throughout the treatment. After the treatment, patients were instructed to avoid strong light exposure and to wear sunglasses, a hat, and long-sleeved clothing if any outdoor activities were required for two weeks, in order to prevent effects of photosensitivity.

### Study design

This study was designed to be conducted in two stages over a total 16-week period. The initial 8-week stage represented the double-blind, placebo-controlled treatment period (stage 1: day 1 to week 8) and aimed to establish efficacy. The subsequent 8-week stage represented the all-treatment period (stage 2: week 8 to week 16) and aimed to assess the overall efficacy and safety profiles.

**Fig 1:**
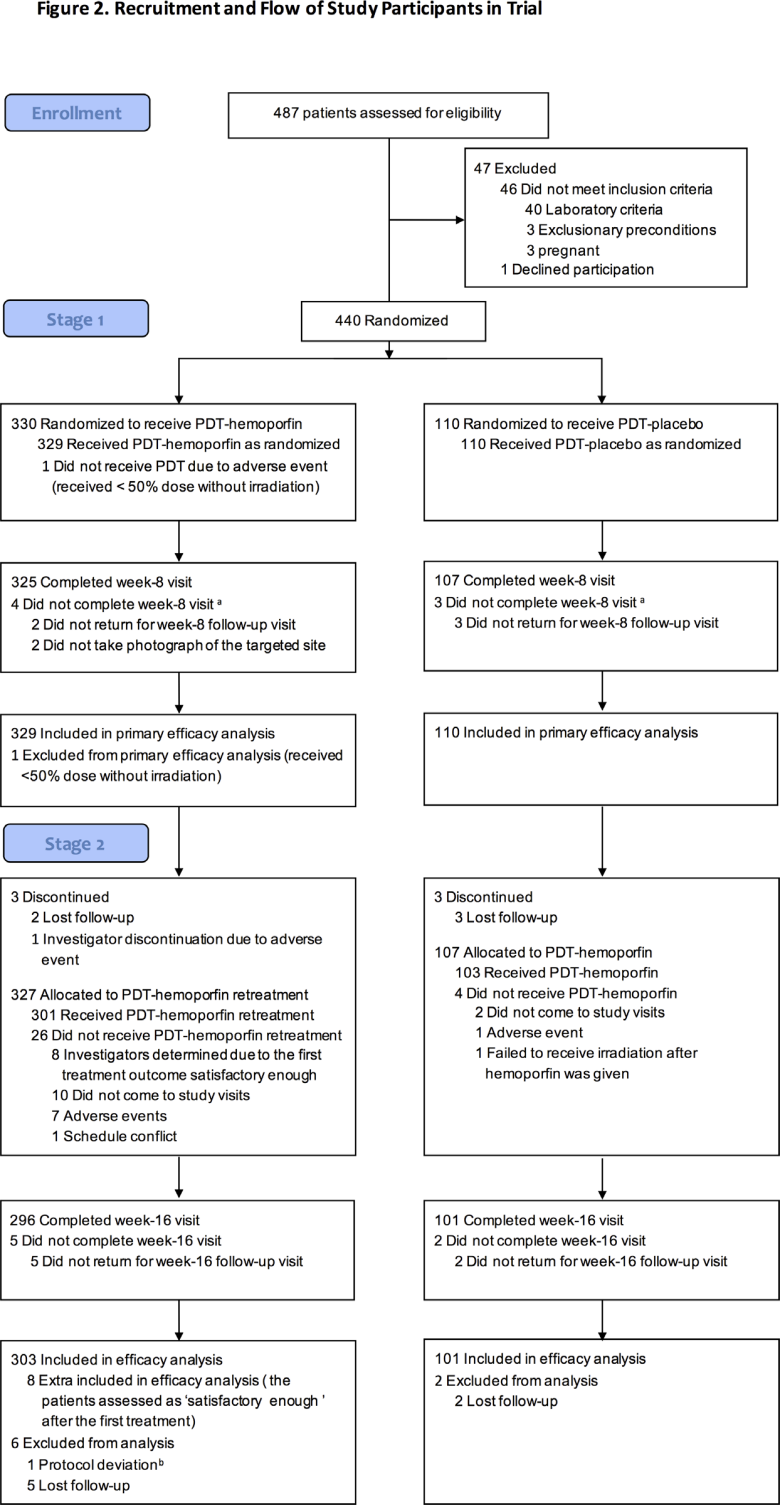
Flowchart of patient enrollment, treatment group allotment, and progression through the study period. Abbreviations: PDT-hemoporfin, hemoporfin-mediated PDT; PDT-placebo, laser irradiation plus placebo. Denotations: ^a^Data from participants were included in the primary efficacy analysis, missing data were imputed as no improvement. ^b^A different area of the targeted lesion was treated.

**Fig 2:**
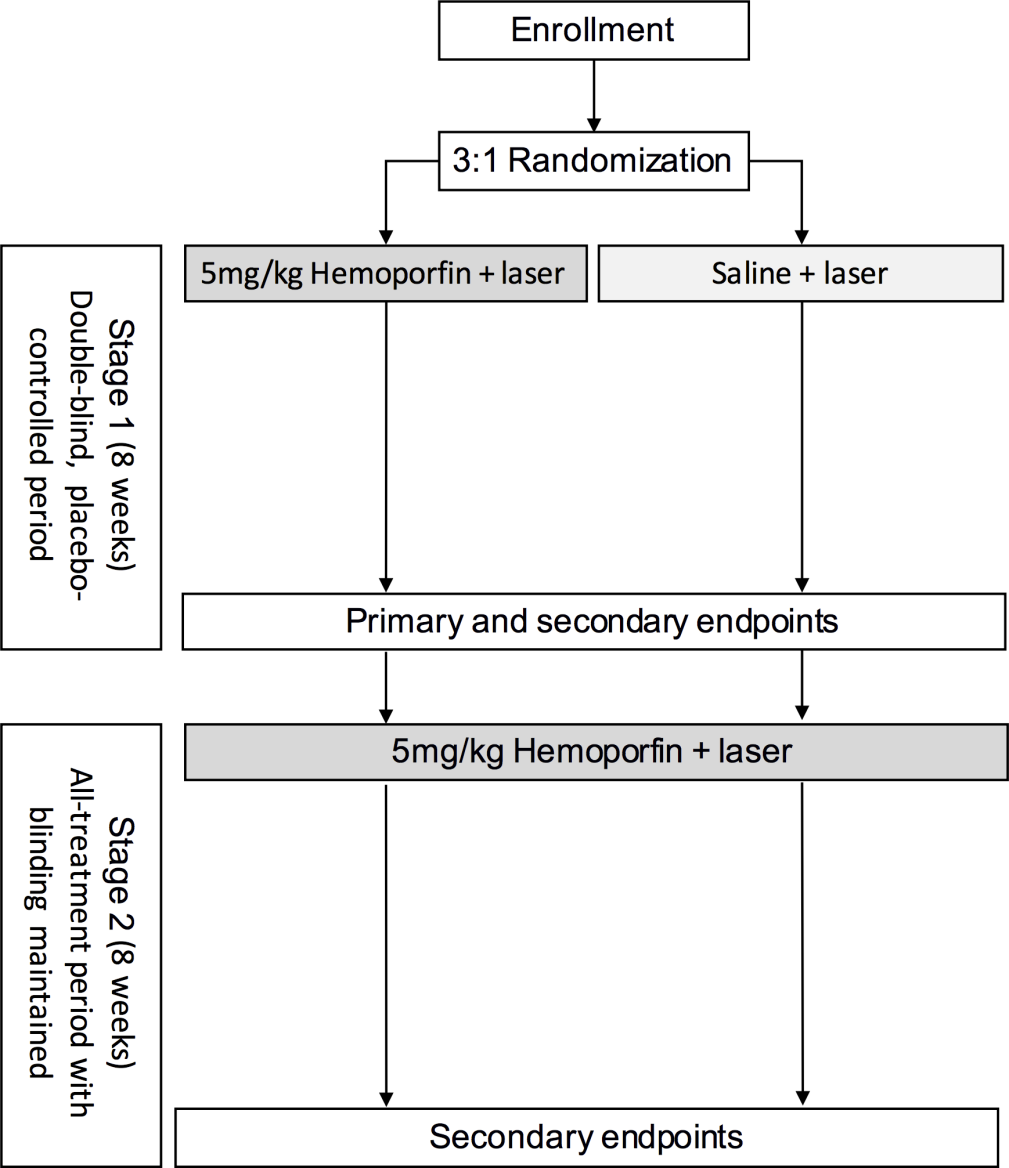
Trial design.

Upon enrollment, patients were randomized at a 3:1 ratio to receive hemoporfin or placebo, respectively, by using a block randomization scheme (block size = 4, 110 blocks) stratified according to investigational site. At day 1 (Stage 1), the patients received laser irradiation with hemoporfin (designated as the PDT-hemoporfin group) or irradiation with placebo (designated as the PDT-placebo group), with both the physician and the patients unaware of the assignment. To ensure all patients received appropriate treatment for their PWS, including those in the placebo group, the study was designed so that at week 8 (stage 2), all the patients received hemoporfin-PDT. In addition to the days of treatment (day 1 and week 8) visits, follow-up visits occurred on post-treatment day 4, 4 days after the week 8 visit, and week 16. If an adequately satisfactory treatment response had been achieved at week 8, the patient could opt to not receive the second treatment. The profile of patients throughout the study is summarized in Fig 1, and the trial design is shown in Fig 2.

### Efficacy analyses

Standardized digital photos of the targeted sites were taken from three different angles (at 90° and at 45° to the left and right of the treated surface) before and after each treatment, and at each visit (standard operating procedure described in the S3 File). Prior to photographing, each PWS lesion was labeled with a marker of red coloration that was used as a reference marker and as a quality control marker in the photographs for subsequent judgment of treatment efficacy. Three blinded evaluators (one dermatologist and two plastic surgeons, who were otherwise not involved in the study) independently reviewed the photos from both stages of the study and graded the extent of PWS fading (improvement) according to color blanching from the baseline in the treated area and using the following four-level scale: no improvement (NI): <20%; some improvement (SI): 20–59%; great improvement (GI): 60–89%; or nearly completely resolved (CR): ≥90%. The results were deemed valid when two or more evaluators agreed; otherwise, the response was re-evaluated until a consensus of two or more was achieved. Initial disagreement in the evaluation occurred for only 4.62% of the patients in the primary efficacy analysis. The primary efficacy endpoint was proportion of patients achieving at least SI at week 8. The secondary efficacy endpoints were proportion of patients achieving CR and at least GI at week 8, proportion of patients achieving CR, at least GI or at least SI at week 16, and corresponding satisfaction of the investigators and the patients themselves, which was designated as ‘excellent’, ‘good’, ‘moderate’, or ‘ineffective’ at weeks 8 and 16.

For post hoc objective efficacy assessment, the digital photos were analyzed as previously described [18,19]. Briefly, after opening in the ImageJ software [20], each photo was converted into an erythema index (EI, representing the intensity of redness) image file. A region of interest (ROI) was selected and its EI was automatically measured. For each image, triplicate ROIs within the PWS lesion (ls) and within adjacent normal skin (ns) were given EI measurements for comparative analysis of the averaged values, respectively. To maintain operational accuracy, we created a macro of the ImageJ software which was then used to process the images.

The EI difference (ΔEI) was calculated as: EI_ls_−EI_ns_. Hence, the ΔEI value represented the difference in the degree of erythema between the PWS lesion and the adjacent normal skin.

### Safety analyses

Clinical assessments by the physicians and all local or systemic events reported by the patients were recorded in detail. The following symptoms were recorded as treatment reactions: local burning sensation, pain, pruritus, numbness, edema, purpura, blistering, and crusting at the treatment site. Laboratory examinations (including routine blood and urine tests, liver and renal function tests, as well as electrocardiograms) were also performed (at baseline, day 4, week 8, and 4 days after week 8) to monitor adverse events. All adverse events and treatment reactions recorded during the 16-week trial period were followed-up until they had completely resolved. The adverse events were coded according to the World Health Organization Adverse Reaction Terminology (WHO-ART, version 2000) [21] and the extent of each adverse event was defined as follows: mild, awareness of a sign or symptom that is otherwise easily tolerable; moderate, discomfort that is sufficient to cause interference with normal activities; severe, incapacitating and inhibiting the ability to perform normal activities. Causality between the study drug and an adverse event was defined using the WHO-UMC causality assessment system [22]. A serious adverse event was defined as any adverse events occurring at any doses that might result in death, threaten the life of the patient, require hospitalization or a prolonged stay in the hospital, cause long-term or significant disability, or cause congenital malformations.

### Statistical analyses

With chi-square test, we calculated that the sample size of 88 patients (66 in the treatment group and 22 in the placebo group) would provide 90% power to detect a 40% difference in the proportion of patients achieving at least SI (improvement ≥20%) at week 8, on the basis of a two-sided significance level of 0.05 and assuming that 75% of the patients in the treatment group would achieve at least SI according to the prior phase II studies [16]. However, considering the minimum sample size of 300 cases that is demanded by official regulatory for safety observation for new drug registration in China and assuming a 10% drop-out rate, we planned to include 440 patients (330 in treatment group and 110 in the placebo group).

Analysis of the primary outcome was conducted with chi-square test in the intent-to-treat (ITT) population (all randomized patients who had received at least one dose of treatment in each group); the seven patients (1.59%; four in the PDT-hemoporfin group and three in the placebo group) with missing post-baseline data were imputed as ‘no improvement’, according to the non-responder imputation approach. Univariate and stepwise multivariate logistic regression analyses were performed to assess the significance of differences in the treatment group and to identify potential confounding factors. Further subgroup analyses were made by the chi-square tests or Fisher’s exact tests (S2 and S3 Tables according to sex, age group (adolescent: 14–18 years old, young adult:19–30 years old, and older adult: 31–65 years old), PWS type (pink, purple, and hypertrophic), and location (centrofacial, non-centrofacial, and neck).

Analysis of the secondary outcomes, including proportion of patients achieving CR or at least GI at week 8, proportion of patients achieving CR, at least GI, or at least SI at week 16, and the satisfaction of the investigators and the patients at weeks 8 and 16, were made using chi-square test or Fisher’s exact test. An additional analysis was performed using *t*-test to compare the changes of ΔEI between two groups at week 8 and 16. Missing data were not imputed for some secondary outcomes. Eight of the patients who had achieved an adequately satisfactory response at week 8 after the initial treatment and opted to not receive a second treatment were imputed in the efficacy analysis at week 16; data from these patients were analyzed according to their original treatment group (all were in the PDT-hemoporfin group).

The safety analysis was based on event incidence rates adjusted for exposure (one treatment of a patient was defined as a patient-treatment). The evaluable-for-safety population consisted of those patients who had received study medication and who had at least one post-baseline safety evaluation. The treatment reactions and adverse events were analyzed separately using chi-square tests or Fisher’s exact test.

All statistical analyses were carried out by the SAS statistical software package (version 9.1.3). All statistical tests were two-sided with a significance (a) level of 0.05.

## Results

### Study patients

The study was conducted from 2008 to 2010. All 440 study participants had skin type III-IV on the Fitzpatrick phototype scale. The PDT-hemoporfin group (*n* = 329) had slightly more males, greater height and weight, and less pink-type PWS lesions than the PDT-placebo group (*n* = 110) (Table 1). 5.0% of the total study population had received prior treatment for PWS with laser, medication, or other therapeutic procedures.

**Table 1:** Patients’ demographics and disease history^†^.

Feature		PDT-hemoporfin	PDT-placebo
		*n* = 329	*n* = 110
Age (years)		24.95±7.80	24.17±6.84
Age group (years, *n*[%])			
	14–18	48(14.6)	13(11.8)
	19–30	220(66.9)	82(74.5)
	31–65	61(18.5)	15(13.6)
Males, *n*(%)		138(41.9)	33(30.0)
Females, *n*(%)		191(58.1)	77(70.0)
Chinese ethnicity, *n*(%)			
	Han	323(98.2)	110(100.0)
	Non-Han	6(1.8)	0(0.0)
Occupation, *n*(%)			
	PL	36(10.9)	15(13.6)
	Non-PL	293(89.1)	95(86.4)
Height (cm)		165.09±7.95	163.39±7.31
Weight (kg)		57.35±9.97	54.67±8.33
Systolic blood pressure (mmHg)		113.01±13.09	112.85±12.07
Diastolic blood pressure (mmHg)		72.85±9.00	73.31±8.69
Heart rate (bpm)		80.38±10.59	80.09±10.82
Previous therapy, *n*(%)		19(5.8)	3(2.7)
Location of PWS, *n*(%)			
	Non-CF	105(31.9)	36(32.7)
	CF	162(49.2)	64(58.2)
	Neck	60(18.2)	9(8.2)
	Other	2(0.6)	1(0.9)
Type of PWS^‡^, *n*(%)			
	Pink	111(33.7)	49(44.5)
	Purple	189(57.4)	56(50.9)
	Hypertrophic	29(8.8)	5(4.5)
Area of targeted site (cm^2^)		34.38±15.81	31.64±16.00
Power density (mW/cm^2)^		86.51±3.27	86.63±3.18
ΔEI at baseline (95% CI)		39.94(38.04 to 41.84)	39.90(36.92 to 42.88)

Abbreviations: PL, physical laborers; CF, centrofacial

Denotations

^†^ Data are presented as means ± SD, unless otherwise specified.

^‡^ Pink type: flat PWS lesion with color of light pink to red, which fades completely upon pressure; Purple type: flat PWS lesion with color of purple to dark purple, which fades completely or incompletely upon pressure; Hypertrophic type: thickened PWS lesion with a nodular or raised surface (above the around normal skin plane), having a dark purple color, which incompletely fades or shows no fade upon pressure.

### Efficacy

**Fig 3:**
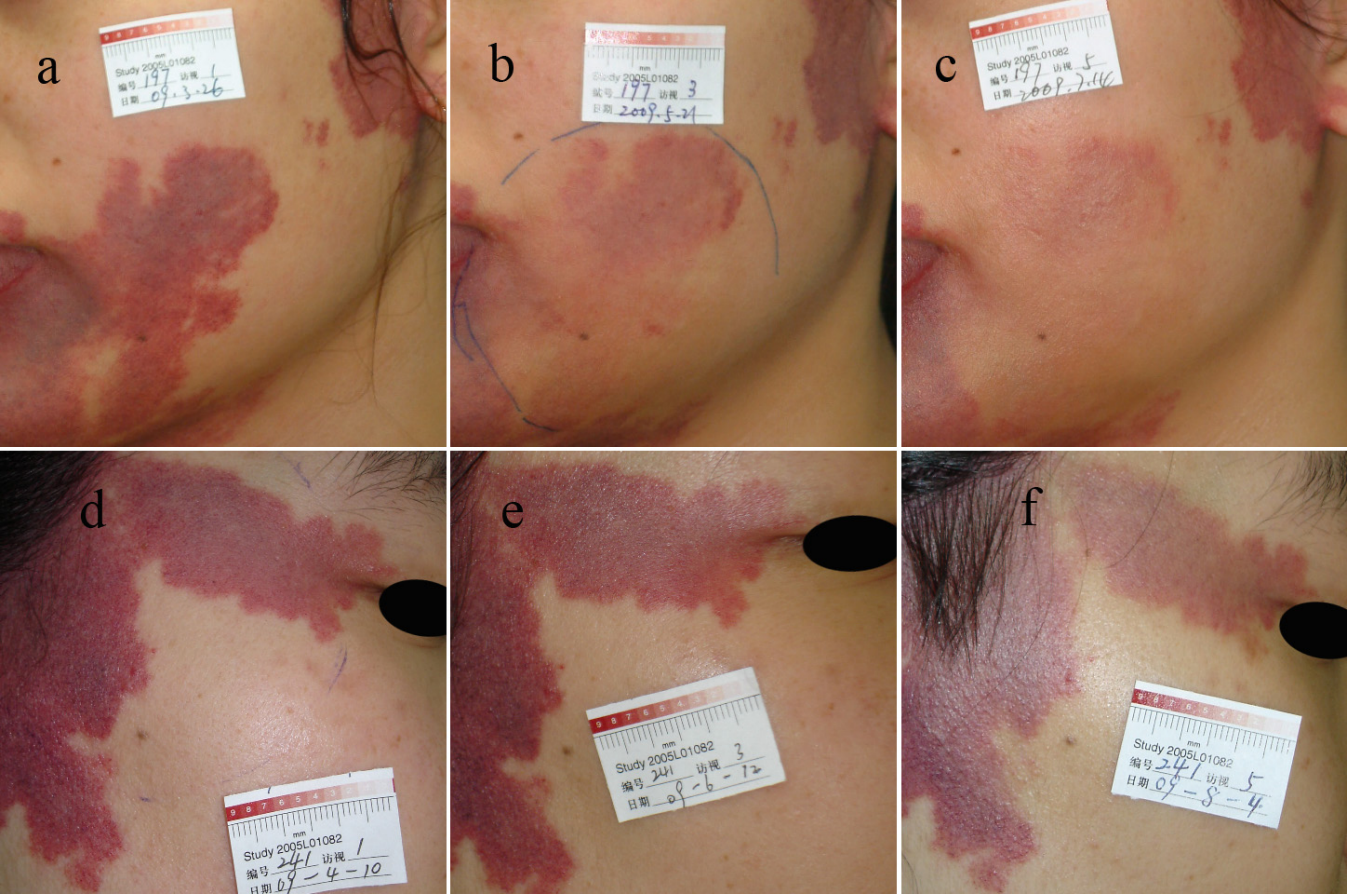
Efficacy of PDT-hemoporfin for treating PWS during the 16-week study period. Panels a, b and c represent a patient from the PDT-hemoporfin group and d, e and f represent a patient from the placebo group; Panels a and d: baseline; b: after 1 PDT-hemoporfin treatment at week 8; c: after 2 PDT-hemoporfin treatments at week 16; e: after PDT-placebo at week 8; f: after 1 PDT-hemoporfin treatment at week 16; Panels c, b, f and e show PWS fading by ≥90%, 60–89%, 20–59% and <20%, respectively.

In total, 439 patients were eligible for primary analysis. (Fig 3A–3F) shows representative images of PWS patients during the 16-week study period. In general, the PDT-hemoporfin group had significantly higher proportions of patients in all response categories at post-treatment week 8 (vs. PDT-placebo: at least SI, 89.7% (*n* = 295; 95% CI, 85.9%-92.5%) vs. 24.5% (*n* = 27; 95% CI, 17.4%-33.3%); at least GI, 43.5% (*n* = 143; 95% CI, 38.2%-48.9%) vs. 0.9% (*n* = 1; 95% CI, 0.2%-5.0%); CR, 11.2% (*n* = 37; 95% CI, 8.3%-15.1%) vs. 0.0% (*n* = 0; 95% CI, 0.0%-3.4%); *P*<0.0005) (Table 2).

**Table 2:** Efficacy assessment at week 8 and 16.

Efficacy assessment	Week 8			Week 16		
	PDT-hemoporfin	PDT-placebo	*P* value	HH	PH	*P* value
***Grading and response rates of PWS fading*, *n(% [95% CI])***						
*N*	329 ^#^	110 ^¶^	-	303^※^	101	-
At least SI (≥20%)	295 (89.7 [85.9 to 92.5])	27 (24.5 [17.4 to 33.3])	<0.0001^a^	295 (97.4 [94.9 to 98.7])	91 (90.1 [82.7 to 94.5])	0.0022
At least GI (≥60%)	143 (43.5 [38.2 to 48.9])	1 (0.9 [0.2 to 5.0])	<0.0001	194 (64.0 [58.5 to 69.2])	43 (42.6 [33.4 to 52.3])	0.0001
CR (≥90%)	37 (11.2 [8.3 to 15.1])	0 (0.0 [0.0 to 3.4])	0.0002	85 (28.1 [23.3 to 33.4])	8 (7.9 [4.1 to 14.9])	<0.0001
GI (60–89%)	106 (32.2 [27.4 to 37.4])	1 (0.9 [0.2 to 5.0])	-	109 (36.0 [30.8 to 41.5])	35 (34.7 [26.1 to 44.3])	-
SI (20–59%)	152 (46.2 [40.9 to 51.6])	26 (23.6 [16.7 to 32.4])	-	101 (33.3 [28.3 to 38.8])	48 (47.5 [38.1 to 57.2])	-
NI (<20%)	34 (10.3 [7.5 to 14.1])	83 (75.5 [66.6 to 82.5])	-	8 (2.6 [1.3 to 5.1])	10 (9.9 [5.5 to 17.3])	-
***ΔEI*, *mean(95% CI)***						
*N*	329 ^#^	110 ^¶^	-	301(2) ^§^	101	-
ΔEI_before_	39.94 (38.04 to 41.84)	39.90 (36.92 to 42.88)	0.9840	-	-	-
ΔEI_after_	29.58 (27.92 to 31.24)	38.42 (34.78 to 42.05)	<0.0001	25.11 (23.35 to 26.88)	31.16 (27.90 to 34.41)	0.0010
***Subjective assessment of efficacy by the physicians*, *n(% [95% CI])***						
*n(missing)*	327(2) ^§^	107(3) ^§^	-	303	101	-
Good to Excellent	231 (70.7 [65.5 to 75.4])	2 (1.9 [0.5 to 6.6])	<0.0001	252 (83.1 [78.5 to 86.9])	61 (60.4 [50.6 to 69.4])	<0.0001
Excellent	82 (25.1)	0 (0.0)	-	121 (39.9)	20 (19.8)	-
Good	149 (45.6)	2 (1.9)	-	131 (43.2)	41 (40.6)	-
Moderate	80 (24.5)	3 (2.8)	-	45 (14.9)	28 (27.7)	-
Ineffective	16 (4.9)	102 (95.3)	-	6 (2.0)	12 (11.9)	-
***Subjective assessment of efficacy by the patients*, *n(% [95% CI])***						
*n(missing)*	327(2) ^§^	108(2) ^§^	-	303	101	-
Good to Excellent	221 (67.6 [62.3 to 72.4])	3 (2.8 [1.0 to 7.9])	<0.0001	245 (80.9 [76.1 to 84.9])	61 (60.4 [50.6 to 69.4])	<0.0001
Excellent	96 (29.4)	0 (0.0)	-	102 (33.7)	17 (16.8)	-
Good	125 (38.2)	3 (2.8)	-	143 (47.2)	44 (43.6)	-
Moderate	80 (24.5)	8 (7.4)	-	48 (15.8)	30 (29.7)	-
Ineffective	26 (8.0)	97 (89.8)	-	10 (3.3)	10 (9.9)	-

Abbreviations: HH, patients who received a second PDT-hemoporfin treatment; PH, patients who received the first PDT-hemoporfin treatment at week 8 (stage 2); NI, no improvement; SI, some improvement; GI, great improvement; CR, nearly completely resolved.

Denotations

^#^ Four missing data included and imputed as”no improvement”

^¶^ Three missing data included and imputed as”no improvement”

^§^ Missing data not included in the *n*

^※^ Eight of the patients achieved ‘adequately satisfactory’ at week 8 and did not receive the second treatment, but were imputed in the efficacy analyses at week 16; among these eight patients, three, four and one patient were graded as having achieved PWS fading of >90%, 60–89%, and 20–59%, respectively

^a^ primary efficacy end point; ΔEI was analyzed using *t*-test and all other comparisons were conducted using chi-square or Fisher’s exact test.

The multivariate logistic regression analysis confirmed that PDT-hemoporfin treatment was significantly related to a higher likelihood of achieving at least SI (OR 29.324, 95% CI 16.490–54.244, P <0.001), with location and type of PWS as influential factors (a lower proportion of the patients having hypertrophic type and a higher proportion of those having lesions on the neck achieved at least SI, Table 3). A significantly greater proportion of the PDT-hemoporfin group was evaluated as ‘good to excellent’ by the investigators (70.7% **[***n* = 231; 95% CI, 65.5%-75.4%] vs. 1.9% [*n* = 2; 95% CI, 0.5%-6.6%]; *P*<0.0001). Similarly, a significantly greater proportion of the PDT-hemoporfin patients assessed their own response as ‘good to excellent’ (67.6% **[***n* = 221; 95% CI, 62.3%-72.4%] vs. 2.8% [*n* = 3; 95% CI, 1.0%-7.9%]; P<0.0001).

**Table 3:** The logistic regression analysis to identify factors relevant to achieving at least SI at week 8^*^.

Variables	Estimated Odds Ratio (95% CI)	*P* value
PDT-hemoporfin vs. PDT-placebo	29.324(16.490,54.244)	<0.001
Location of PWS (vs. Centrofacial)		
Non-centrofacial	1.107(0.603,2.052)	0.743
Neck	4.030(1.475,12.629)	0.010
Other	1.059(0.053,39.141)	0.973
Type of PWS (vs. Pink type)		
Purple type	1.140(0.617,2.092)	0.673
Hypertrophic type	0.354(0.137,0.951)	0.035

Abbreviations: SI, some improvement; CI, confidence interval.

Denotations

^*^ Candidate continuous variables considered were age, systolic blood pressure (mmHg), diastolic blood pressure (mmHg), heart rate (bpm), body-mass index, area of targeted site (cm2), ΔEI at baseline and power density of laser irradiation (mW/cm2). Candidate categorical variables considered were intervention group (PDT-hemoporfin vs. PDT-placebo), study site (other sites vs. site 1), sex (male vs. female), occupation (physical laborers vs. non-physical laborers), location of PWS (other locations vs. centrofacial) and type of PWS (other types vs. pink type). The variables with significance level of *P* < 0.2 in univariate analyses were included in the stepwise multivariate logistic regression; ethnicity and previous therapy was not included in the analyses because of the small number of cases in the subgroups. The logistic regression model fit was tested with the likelihood ratio test (*P* < 0.001).

Patients in the PDT-hemoporfin group who received a second treatment (*n* = 303) showed significantly higher proportions of response (in all response categories) at week 16 (vs. PDT-placebo group after one treatment with PDT-hemoporfin (*n* = 101): at least SI, 97.4% (*n* = 295; 95% CI, 94.9%-98.7%) vs. 90.1% (*n* = 91; 95% CI, 82.7%-94.5%); at least GI, 64.0% (*n* = 194; 95% CI, 58.5%-69.2%) vs. 42.6% (*n* = 43; 95% CI, 33.4%-52.3%); CR, 28.1% (*n* = 85; 95% CI, 23.3%-33.4%) vs. 7.9% (*n* = 8; 95% CI, 4.1%-14.9%); *P*<0.005). At week 16, compared with the PDT-placebo patients after one treatment with PDT-hemoporfin, a significantly greater proportion of the PDT-hemoporfin patients who received a second treatment were evaluated as having ‘good to excellent’ response by the investigators (83.1% [*n* = 252; 95% CI, 78.5%-86.9%] vs. 60.4% [*n* = 61; 95% CI, 50.6%-69.4%], *P*<0.0001), and so were by the patients (80.9% [*n* = 245; 95%CI, 76.1%-84.9%] vs. 60.4% [*n* = 61; 95%CI, 50.6%-69.4%], *P*<0.0001).

In post hoc analyses, the ΔEI was found to not be significantly different between the PDT-hemoporfin group (*n* = 329) and the PDT-placebo group (*n* = 110) at baseline (39.94 [95% CI, 38.04–41.84] vs. 39.90 [95%CI, 36.92–42.88]; *P* = 0.984). However, at week 8, the ΔEI of the PDT-hemoporfin group was significantly lower (vs. PDT-placebo group: 29.58 [95% CI, 27.92–31.24] vs. 38.42 [95% CI, 34.78–42.05]; *P*<0.0001). At week 16, the PDT-hemoporfin group who received a second treatment (*n* = 301) had significantly lower ΔEI than the patients in the PDT-placebo group who had received only one PDT-hemoporfin treatment (*n* = 101) (25.11 [95% CI, 23.35–26.88] vs. 31.16 [95% CI, 27.90–34.41]; *P* = 0.0010) (S1 Fig and Table 2).

### Safety

The exposure-adjusted rates of treatment reactions and adverse events are presented in Table 4. Treatment reactions (at stage 1 and/or stage 2) occurred in 99.5% (*n =* 731, 95% CI, 98.7%-99.8%) of the PDT-hemoporfin treatments (*n =* 735 in total), compared to only 39.1% (*n =* 43, 95% CI, 30.5%-48.4%) of the PDT-placebo treatments (*n* = 110) and the difference was statistically significant (*P*<0.0001). The median number of treatment reactions per patient-treatment was also significantly higher in the PDT-hemoporfin treatments than in the PDT-placebo treatments (4 [range: 1–7] vs. 1 [range: 1–4]; *P*<0.0001, Mann-Whitney test). All the treatment reactions reported in the PDT-placebo treatments were mild to moderate. In contrast, the patients who received PDT-hemoporfin experienced the appreciable amounts of severe treatment reactions, including: pain (19.9% [*n* = 142; 95% CI, 17.1%-23.0%]), burning sensation (12.1% [*n* = 71; 95% CI, 9.7%-15.0%]), pruritus (4.6% [*n* = 18; 95% CI, 2.9%-7.2%]), edema (25.6% [*n* = 179; 95% CI, 22.5%-29.0%]), crusting (4.8% [*n* = 24; 95% CI, 3.2%-7.1%]), purpura (0.0% [*n* = 0; 95% CI, 0.0%-5.2%]), and vesicle rash (2.0% [*n* = 1; 95% CI, 0.4%-10.4%]). No significant difference was seen for most of the treatment reactions that were experienced in stage 1 and stage 2 by the patients who received the PDT-hemoporfin treatments, with the notable exceptions of a lower percentage of crusting (73.3% [*n* = 242; 95% CI, 68.3%-77.8%] vs. 58.8% [*n* = 177; 95% CI, 53.2%-64.2%]; *P* = 0.0002) and a higher percentage of edema (93.0% [*n* = 307; 95% CI, 89.7%-95.3%] vs. 96.7% [*n* = 291; 95% CI, 94.0%-98.2%]; *P* = 0.0130).

**Table 4:** Treatment reactions and adverse events possibly related to the treatment.

Event		PDT-hemoporfin, 735 PT of 434 patients			PDT-placebo, 110 PT of 110 patients			*P* value
		*n*	Event rate^†^	Days to resolve^‡^	*n*	Event rate^†^	Days to resolve^‡^	
**Treatment reactions**								
	Pain	713	97.0 (95.5 to 98.0)	0–19	7	6.4 (3.1 to 12.6)	0–2	<0.0001
	Burning sensation	589	80.1 (77.1 to 82.9)	0–10	32	29.1 (21.4 to 38.2)	0–4	<0.0001
	Pruritus	388	52.8 (49.2 to 56.4)	0–47	12	10.9 (6.4 to 18.1)	0–9	<0.0001
	Numbness	1	0.1 (0.0 to 0.8)	0	0	0.0 (0.0 to 3.4)	-	>0.999
	Edema	698	95.0 (93.1 to 96.3)	0–21	4	3.6 (1.4 to 9.0)	0–4	<0.0001
	Crusting	496	67.5 (64.0 to 70.8)	0–46	0	0.0 (0.0 to 3.4)	-	<0.0001
	Purpura	70	9.5 (7.6 to 11.9)	2–15	0	0.0 (0.0 to 3.4)	-	0.0007
	Blistering	51	6.9 (5.3 to 9.0)	1–24	1	0.9 (0.2 to 5.0)	3	0.0139
**Adverse events**								
**Total adverse events**		241	32.8 (29.5 to 36.3)	-	8	7.3 (3.7 to 13.7)	-	<0.001
**Light-exposure related reactions**		10	1.4 (0.7 to 2.5)	-	2	1.8 (0.5 to 6.4)	-	0.955
	Dyspnea, rash and photophobia	1	0.1 (0.0 to 0.8)	0	0	0.0 (0.0 to 3.4)	-	-
	Urticaria	1	0.1 (0.0 to 0.8)	0	0	0.0 (0.0 to 3.4)	-	-
	Photosensitive cheilitis	1	0.1 (0.0 to 0.8)	8	0	0.0 (0.0 to 3.4)	-	-
	Photosensitive dermatitis	0	0.0 (0.0 to 0.5)	-	1	0.9 (0.2 to 5.0)	9	-
	Dizziness and photophobia	5	0.7 (0.3 to 1.6)	0–21	0	0.0 (0.0 to 3.4)	-	-
	Photophobia	2	0.3 (0.1 to 1.0)	4–18	1	0.9 (0.2 to 5.0)	5	-
**Local adverse events**								
**Hyperpigmentation**		168	22.9 (20.0 to 26.0)	16–379^a^	0	0.0 (0.0 to 3.4)	-	<0.001
**Hypopigmentation**		14	1.9 (1.1 to 3.2)	27–268^b^	0	0.0 (0.0 to 3.4)	-	0.288
**Temporary skin lesions**		8	1.1 (0.6 to 2.1)		0	0.0 (0.0 to 3.4)	-	0.566
	Rash maculo-papular	3	0.4 (0.1 to 1.2)	0–3	0	0.0 (0.0 to 3.4)	-	-
	Exudation	1	0.1 (0.0 to 0.8)	2	0	0.0 (0.0 to 3.4)	-	-
	Eczema	1	0.1 (0.0 to 0.8)	14	0	0.0 (0.0 to 3.4)	-	-
	Scaling	1	0.1 (0.0 to 0.8)	9	0	0.0 (0.0 to 3.4)	-	-
	Erythema	1	0.1 (0.0 to 0.8)	10	0	0.0 (0.0 to 3.4)	-	-
	Textural change	1	0.1 (0.0 to 0.8)	105	0	0.0 (0.0 to 3.4)	-	-
**Wound infection**		8	1.1 (0.6 to 2.1)	2–12^c^	0	0.0 (0.0 to 3.4)	-	0.566
**Atrophic scar**		4	0.5 (0.2 to 1.4)	48–208^d^	0	0.0 (0.0 to 3.4)	-	>0.999
**Systemic adverse events**								
**Liver and biliary system disorders** ^e^		8	1.1 (0.6 to 2.1)		2	1.8 (0.5 to 6.4)		0.854
	ALT, AST and TBil increase	1	0.1 (0.0 to 0.8)	52	1	0.9 (0.2 to 5.0)		-
	ALT and AST increase	3	0.4 (0.1 to 1.2)	28–119	0	0.0 (0.0 to 3.4)	-	-
	ALT increase	1	0.1 (0.0 to 0.8)	3	1	0.9 (0.2 to 5.0)		-
	AST increase	2	0.3 (0.1 to 1.0)	6–7	0	0.0 (0.0 to 3.4)	-	-
	TBil increase	1	0.1 (0.0 to 0.8)	54	0	0.0 (0.0 to 3.4)	-	-
**Gastro-intestinal system disorders**		6	0.8 (0.4 to 1.8)	0–1	0	0.0 (0.0 to 3.4)	-	>0.999
	Nausea	5	0.7 (0.3 to 1.6)	0–1	0	0.0 (0.0 to 3.4)	-	-
	Vomiting	1	0.1 (0.0 to 0.8)	0	0	0.0 (0.0 to 3.4)	-	-
**Cardiac disorders**		4	0.5 (0.2 to 1.4)	47–154	1	0.9 (0.2 to 5.0)	51	0.504
	Ventricular arrhythmia	1	0.1 (0.0 to 0.8)	154	0	0.0 (0.0 to 3.4)	-	-
	Conduction disorder	1	0.1 (0.0 to 0.8)	47	1	0.9 (0.2 to 5.0)	51	-
	Non-specific ECG abnormal	2	0.3 (0.1 to 1.0)	47–50	0	0.0 (0.0 to 3.4)	-	-
**Respiratory system disorders**		3	0.4 (0.1 to 1.2)	0–2	0	0.0 (0.0 to 3.4)	-	>0.999
	Dyspnea	3	0.4 (0.1 to 1.2)	0–2	0	0.0 (0.0 to 3.4)	-	-
**General symptoms**		3	0.4 (0.1 to 1.2)	2–36	0	0.0 (0.0 to 3.4)	-	>0.999
	Asthenia	2	0.3 (0.1 to 1.0)	2–30	0	0.0 (0.0 to 3.4)	-	-
	Sweating increased	1	0.1 (0.0 to 0.8)	36	0	0.0 (0.0 to 3.4)	-	-
**Eye abnormality**		2	0.3 (0.1 to 1.0)	4–16	0	0.0 (0.0 to 3.4)	-	>0.999
	Conjunctivitis	1	0.1 (0.0 to 0.8)	16	0	0.0 (0.0 to 3.4)	-	-
	Xerophthalmia	1	0.1 (0.0 to 0.8)	4	0	0.0 (0.0 to 3.4)	-	-
**White cell and RES disorders**		1	0.1 (0.0 to 0.8)	2	0	0.0 (0.0 to 3.4)	-	>0.999
	Leukocytosis	1	0.1 (0.0 to 0.8)	2	0	0.0 (0.0 to 3.4)	-	-
**Nervous system disorders**		1	0.1 (0.0 to 0.8)	7	1	0.9 (0.2 to 5.0)	2	0.244
	Dizziness	0	0.0 (0.0 to 0.5)	-	1	0.9 (0.2 to 5.0)	2	-
	Vertigo	1	0.1 (0.0 to 0.8)	7	0	0.0 (0.0 to 3.4)	-	-
**Tooth disorder**		1	0.1 (0.0 to 0.8)	0	0	0.0 (0.0 to 3.4)	-	>0.999
	Tooth infection	1	0.1 (0.0 to 0.8)	0	0	0.0 (0.0 to 3.4)	-	-
**Urinary system disorders**		0	0.0 (0.0 to 0.5)	-	2	1.8 (0.5 to 6.4)	52–54	0.017
	Hematuria	0	0.0 (0.0 to 0.5)	-	1	0.9 (0.2 to 5.0)	54	-
	Proteinuria	0	0.0 (0.0 to 0.5)	-	1	0.9 (0.2 to 5.0)	52	-

Abbreviations: PT, patient-treatments; AST, aspartate transaminase; ALT, alanine aminotransferase; TBil, total bilirubin; ECG, electrocardiogram; RES, reticuloendothelial system.

Denotations

^†^ Exposure adjusted event rate per 100 patient-treatments, Mean (95% CI)

^‡^ range, where 0 indicates resolution in less than one day

^a^ Nine patients lost to follow-up

^b^ Two patients lost to follow-up

^c^ Healed with application of topical antibiotics

^d^ One patient lost to follow-up

^e^ An increase was defined as ALT or AST exceeding the upper limit of normal (ULN), or TBil exceeding 1.5ULN; All statistical comparisons were conducted using chi-square or Fisher’s exact test.

None of the study participants experienced any treatment related serious adverse events. Only two types of the adverse events reported differed significantly between the patients who received PDT-hemoporfin treatments and the PDT-placebo treatments; in particular, the PDT-hemoporfin treatments were associated with a significantly higher proportion of hyperpigmentation (22.9 [95% CI, 20.0–26.0] vs. 0.0 [95% CI, 0.0–3.4] per 100 patient-treatment; *P*<0.001) and a significantly lower rate of urinary system disorder (0.0 [95% CI, 0.0–0.5] vs. 1.8 [95%CI, 0.5–6.4] per 100 patient-treatments; *P* = 0.017). All the adverse events were mild to moderate, except for 2.4% (95% CI, 1.5%-3.8%) of the hyperpigmentation that were severe. All the adverse events resolved without sequelae during follow-up; however, the final outcome of 12 adverse events remains unknown since those patients were lost of follow-up before the event had been fully resolved. All rates of adverse events in the PDT-hemoporfin group were similar between stage 1 and stage 2.

## Discussion

Ideally, PWS should be treated in a manner that provides the best cosmetic outcome. To achieve this goal, innovative approaches in PDT need to be developed, possibly at the levels of drug as well as and the treatment protocol. Hemoporfin is a new product of the porphyrin-related photosensitizer HMME, and has been shown to elicit a stronger photodynamic effect, lower toxicity, and a shorter-term skin phototoxicity than the most commonly used photosensitizer, photofrin [16,23,24]. The protocol used in the current study, which was designed according to the results from our exploratory phase IIa [16] and IIb studies to identify an optimized irradiation procedure and drug dosage, is novel. However, the effectiveness and safety of this protocol remains to be established by a prospective study of a large population. To our knowledge, this is the first randomized clinical trial of a large sample population to assess the efficacy and safety of PDT-hemoporfin for treating PWS in adolescents and adults. The stage 1 was designed as randomized double-blind, placebo-controlled, to establish efficacy; and in stage 2, all patients in both group were included for treatment, for a better safety evaluation and ethical practice.

### Efficacy profile of PDT-hemoporfin for treating PWS

Although it is possible to quantify PWS lesion response to treatment by colorimetry or reflectance spectrophotometry [25], the clinical utility of these techniques is limited by a lack of repeatability [18,26]. However, use of a defined scoring system based on comparative analysis of pre- and post-treatment images has been shown to be a valid method for assessing treatment response of PWS, and has been successfully applied to other clinical studies, including randomized clinical trials [27–29]. This method was applied in the current study, and indicated that at post-treatment week 8 a statistically significant and clinically meaningful reduction in disease severity of PWS had occurred in patients who underwent PDT-hemoporfin treatment, as opposed to the placebo-control group who underwent laser irradiation without hemoporfin.

Previous studies have suggested that efficacy of PDT might be affected by sex and age, as well as PWS location and type (the pink, purple and hypertrophic type is relevant to lesion severity of PWS), presumably due to variations in the lesion-involved skin and vessel properties [30,31]^.^ The current study also found that patients who were female and having cervical and pink type PWS responded better to the PDT-hemoporfin treatment (Table 3, S2 and S3 Tables).

The higher proportions of physician- and patient-rated satisfaction with treatment response in the PDT-hemoporfin group, and after the second treatment application, suggest that multiple treatment sessions may be preferable for PWS. EI analysis has been previously used to quantitatively evaluate the intensity of erythema [18] and the efficacy of treatment for PWS [19]. Indeed, in post hoc analyses, the significantly lower ΔEI that was achieved after a single PDT-hemoporfin treatment (vs. the PDT-placebo treatment) was even further reduced after a second application of the procedure.

Alternative treatments exist for PWS, and many have been systematically studied as well. For example, in a comparative analysis of PDT and pulsed-dye laser (PDL) in a small series of PWS patients, PDT was shown to be at least as effective as PDL, and in some cases to be superior [14,32]. It has been suggested that PDT might be effective in treating PDL-resistant PWS lesions [33], or that a combination PDT plus PDL treatment strategy may provide better results for PWS patients [34]; however, these possibilities must be assessed in future studies.

### Safety profile of PDT-hemoporfin for treating PWS

Treatment reactions were observed in almost all of the patient-treatments of the current study. While the rates of treatment reactions in the PDT-placebo treatments were lower and less severe than those in the PDT-hemoporfin treatments, the fact that these reactions occurred indicates the potential of the laser irradiation component of the procedure causing some of the treatment reactions. Assessment of the most frequently experienced treatment reactions provided insights into the possible management of these undesirable side effects. For example, in the case of pain, the symptom usually began around 5–10 minutes after the initiation of laser exposure and blowing cold air on the area during the treatment process might help to ease the discomfort [35]. The patients who received a second PDT-hemoporfin treatment had different incidences of some treatment reactions, namely crusting and edema. PDT-induced microstructural changes in the treated skin may play a role in this phenomenon [36,37], but further study is needed to determine the underlying mechanism.

Our treatment showed similar rates of treatment reaction, but some different rates of long term side effects compared to a retrospective study. The differences might be owing to study design, treatment protocol or methods of observation for AEs [38]. Transient hyperpigmentation was the principal adverse event associated with the PDT-hemoporfin treatment in this study. PDT is limited by its induction of prolonged systemic photosensitivity to visible light, which can last for 1~2 months following application of porphyrin derivatives, such as photocarcinorin [11,39]. However, in the current study, when simple and convenient protection practices were used over the 2 week period following the treatment procedure, the incidence of light-exposure related reactions were not significantly increased in the PDT-hemoporfin treated patients. Thus, the patient might be allowed to cautiously resume normal daily activities shortly after treatment.

Although fine tuning of drug dose or exposure time according to selected observation parameters (e.g. age, sex, PWS type and location, or changes of the skin during irradiation) might be helpful to improve the efficacy of the treatment and reduce the adverse effects, it would hard for practice and might be risky without known the risk/benefit profile defined in the population. Our study has settled the basis of this possibility by define the risk/benefit profile of an optimized protocol. In our study, PWS patients treated with PDT using our protocol achieved satisfactory efficacy at the risk of only short-term treatment reactions and partly temporary hyperpigmentation, no significant scar or systemic side effects. Thus the risk/benefit profile of the treatment was remarkable. Retrospective or small scale studies have been reported to compare the efficacy and safety profiles of PDT with those of PDL [14,32,40]. The rate of excellent response in PDT group was significantly higher than that in PDL group (23.5–37.5% vs 3.1–16.1%), and incidences of pigmentation and scar formation in PDT group were significantly lower than PDL group (8.3–10.2% vs 21.1–24.7%) [14, 40]. A significantly greater blanching effect of PWS has been shown after a single-session PDL treatment compared with a single-session PDT treatment [32]. However, the real risk/benefit difference between PDT and PDL is necessary to be studied in large scale prospective studies.

### Limitations

This study had several limitations. Firstly, the current study did not attempt to compare the effects of PDT with PDL. Because large multicenter studies to compare the efficacies of these two treatments have, unfortunately, been unfeasible at our settings due to the limitations of PDL being more operator-dependent than PDT and other practical reasons. Secondly, in practice, the efficacy of treatment for PWS will be stable following resolution of acute responses; thus, the efficacy at week 16 likely represents the response over the period of upcoming years [30]. However, as the alternative PWS treatment of PDL is associated with significant recurrence or redarkening of the treated lesion during long-term follow-up [7,8], the long-term efficacy of PDT-hemoporfin should also be evaluated in further studies. Lastly, the half-life of intravenous-administered hemoporfin is short (1.31 ± 0.33h [16]); therefore, we assume that the observation period of 8 weeks used in each stage of this study was likely sufficient for monitoring adverse events related to the treatment. However, longer-term (>16 weeks) monitoring of PDT-hemoporfin-treated patients is necessary to help identify very rare adverse events. Further randomized controlled trials to evaluate the effect in comparisons with PDL and the long term efficacy and safety are in planning.

## Conclusions

Hemoporfin-mediated PDT using 5mg/kg hemoporfin and 532 nm continuous wave lasers with fluence of 96–120 J/cm^2^ is effective and safe for adolescent and adult patients with port-wine stain.

## Supporting Information

S1 Fig Representative erythema index images. (PDF)

S1 File Consort Checklist. (DOCX)

S2 File Study Protocol. (PDF)

S3 File Standard operation procedure for digital photography used in this study. (DOCX)

S1 Table Details of laser instruments used in this study. (DOCX)

S2 Table Stratified analysis of efficacy at week 8 for the patients who received hemoporfin in stage 1. (DOCX)

S3 Table Stratified analysis of efficacy based on PWS type at week 8. (DOCX)
