# Supplementary material for: Hemoporfin Photodynamic Therapy for Port-Wine Stain: A Randomized Controlled Trial
Source: PLoS One. 2016 May 26;11(5):e0156219. doi: 10.1371/journal.pone.0156219 (PMC4881994; doi:10.1371/journal.pone.0156219)
Supplement: S1 Fig — (PDF) [file pone.0156219.s001.pdf]

S1 Fig. Representative erythema index images

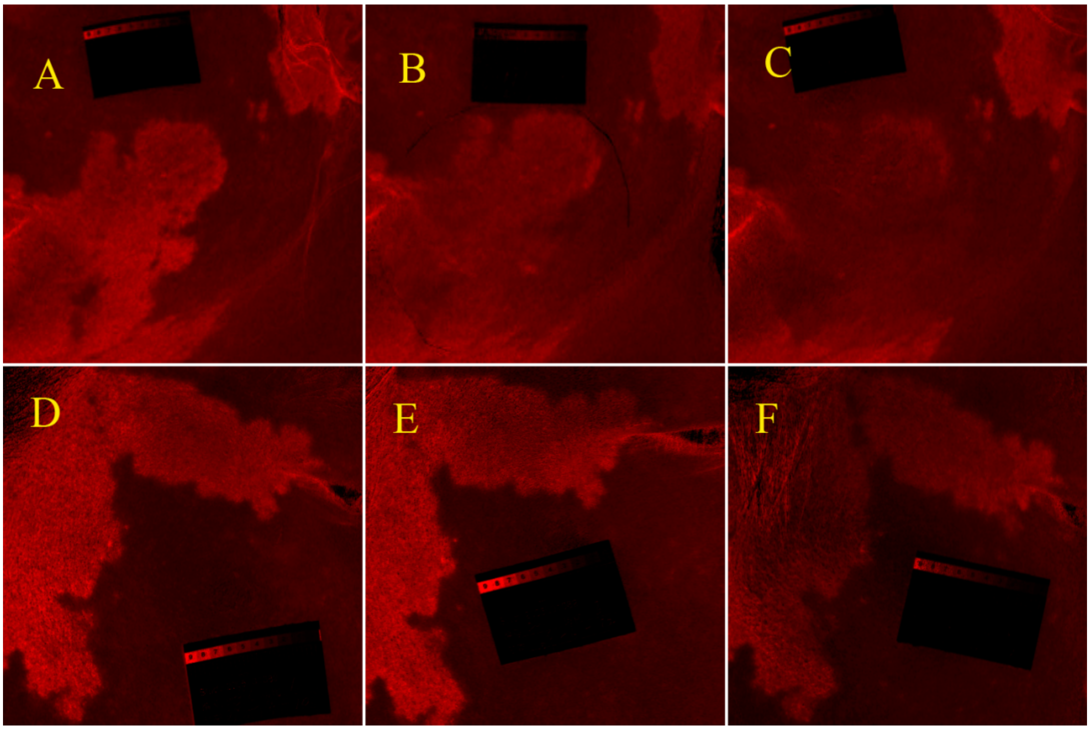

These pictures were from the same two patients as presented in Figure 2. Panels A, B and C represent a patient from the PDT-hemoporfin group and D, E and F represent a patient from the placebo group; Panels A and D: baseline; B: after 1 PDT-hemoporfin treatment at week 8; C: after 2 PDT-hemoporfin treatments at week 16; E: after PDT-placebo at week 8; F: after 1 PDT-hemoporfin treatment at week 16; Panels C, B, F and E show PWS fading by $\geq$90%, 60-89%, 20-59% and <20%, respectively. ΔEI: A, 76.8; B, 43.3; C, 29.0; D,54.4; E,57.7; F, 39.0.
