# Supplementary material for: Hemoporfin Photodynamic Therapy for Port-Wine Stain: A Randomized Controlled Trial
Source: PLoS One. 2016 May 26;11(5):e0156219. doi: 10.1371/journal.pone.0156219 (PMC4881994; doi:10.1371/journal.pone.0156219)
Supplement: S2 File — (PDF) [file pone.0156219.s003.pdf]

# Hemoporfin for Injection Phase III Clinical Trial Protocol

(Protocol No. HMME-08-12)

| | |
|---|---|
| **Study title:** | A multicenter clinical trial (phase III) to evaluate the efficacy and safety of hemoporfin for injection for the treatment of port-wine stains |
| **Sponsor:** | Shanghai Fudan-Zhangjiang Bio-Pharmaceutical Co., Ltd.<br>Chinese PLA General Hospital |
| **Clinical institution:** | Peking University First Hospital<br>Institute of Dermatology, Chinese Academy of Medical Sciences<br>The Ninth People's Hospital of Shanghai Jiaotong University School of Medicine<br>Guangzhou General Hospital of Guangzhou Military Region<br>Huashan Hospital of Fudan University<br>Xijing Hospital of the Fourth Military Medical University<br>Union Hospital of Tongji Medical College, Huazhong University of Science and Technology<br>Xiangya Hospital of Central South University |
| **Statistical institution:** | Drug Clinical Research Center of Shanghai University of Traditional Chinese Medicine |
| **Principal investigator:** | Department of Dermatology and Venereology, Peking University First Hospital<br>Xuejun Zhu Professor |

**Clinical study plan date: Sep. 2008 – Dec. 2009**

**Confidential Document**

Importance: this document contains confidential materials of **Shanghai Fudan-Zhangjiang Bio-Pharmaceutical Co., Ltd.** and **Chinese PLA General Hospital**, which should not be used, disclosed or published without authorization of **Shanghai Fudan-Zhangjiang Bio-Pharmaceutical Co., Ltd.** and **Chinese PLA General Hospital**.

Signing this clinical protocol to confirm that: I have carefully read and understood all the articles of this study protocol entitled "**A multicenter clinical trial to evaluate the efficacy and safety of hemoporfin for injection for the treatment of port-wine stains**", and I am prepared to carry out this study in strict compliance with the requirements of this study protocol. Meanwhile, I understand that any changes made to this study protocol must be approved in written by the sponsor in advance.

| | | |
|---|---|---|
| Zhu Xuejun<br>Principal investigatior<br>Department of Dermatology and Venereology, Peking University First Hospital | Signature | Date |
| Tu Ping<br>Investigator<br>Department of Dermatology and Venereology, Peking University First Hospital | Signature | Date |
| Zhou Zhanchao<br>Investigator<br>Institute of Dermatology, Chinese Academy of Medical Sciences<br>Laser Center | Signature | Date |
| Zhou Guoyu<br>Investigator<br>The Ninth People's Hospital of Shanghai Jiaotong University School of Medicine<br>Department of Oral and Maxillofacial Surgery | Signature | Date |
| Lin Xiaoxi<br>Investigator<br>Department of Plastic and Reconstructive Surgery | Signature | Date |
| Yang Huilan<br>Investigator<br>Department of Dermatology, Guangzhou General Hospital of Guangzhou Military Region | Signature | Date |

Signing this clinical protocol to confirm that: I have carefully read and understood all the articles of this study protocol entitled "**A multicenter clinical trial to evaluate the efficacy and safety of hemoporfin for injection for the treatment of port-wine stains**", and I am prepared to carry out this study in strict compliance with the requirements of this study protocol. Meanwhile, I understand that any changes made to this study protocol must be approved in written by the sponsor in advance.

| | | |
|---|---|---|
| Xu Jinhua<br>Investigator<br>Department of Dermatology and Venereology, Huashan Hospital of Fudan University | Signature | Date |
| Gao Tianwen<br>Department of Dermatology, Xijing Hospital of the Fourth Military Medical University | Signature | Date |
| Tu Yating<br>Investigator<br>Department of Dermatology and Venereology, Union Hospital of Tongji Medical College, Huazhong University of Science and Technology | Signature | Date |
| Xie Hongfu<br>Investigator<br>Department of Dermatology and Venereology, Xiangya Hospital of Central South University | Signature | Date |
| Zheng Qingshan<br>Principal statistician<br>Drug Clinical Research Center of Shanghai University of Traditional Chinese Medicine | Signature | Date |
| Tao Jining<br>Sponsor:<br>Shanghai Fudan-Zhangjiang Bio-Pharmaceutical Co., Ltd. | Signature | Date |

## A multicenter clinical trial (phase III) to evaluate the efficacy and safety of hemoporfin for injection for the treatment of port-wine stains

1. Trial background

Port-wine stains, also known as nevus flammeus or nevus telangiectasia, can occur in any part of the body, more frequently in the face, neck and scalp, affecting the appearance and psychology of patients. Port-wine stains is malformation of the superficial dermal capillaries, the essential difficulty in treatment is to remove the dilated superficial dermal capillaries to eliminate the abnormal red color of the lesion area without damaging the epidermis and deep dermal tissues in order to achieve a scar-free therapeutic goal. Treatments including cryosurgery, excision followed by skin grafting, X-ray, isotope and laser either leaves scars or is unable to eliminate color of the lesion area completely. Therefore, it is a clinical challenge to develop highly selective therapeutic methods.

Photodynamic therapy (PDT) is a therapeutic method that employs photosensitizers to generate cytotoxic substances after irradiation with visible light of specific wavelengths to act on the target tissues and produce histological effect. The photosensitizers can absorb photons and pass the energy to oxygen molecules to induce chemical reactions producing singlet oxygen and other toxic substances. An important property of photosensitizers is the ability to concentrate preferentially in the lesional tissues to produce specific biological effects with minimal impact on the surrounding normal tissues. Currently, PDT has demonstrated remarkable efficacy in the treatment of condyloma acuminate, actinic keratosis and other diseases.

The mechanism of PDT for the treatment of port-wine stains: because the photosensitizer obtains a plasma concentration peak immediately after its intravenous injection followed by rapid absorption by the vascular endothelial cells with little absorption in the epidermal cells, the distribution of injected photosensitizer forms an apparent concentration gradient between the vascular endothelial cells and the epidermal cells. Then transepidermal laser irradiation using specific wavelengths of selective absorption can activate the photosensitizers to generate singlet oxygen and other phototoxic substances, leading to selective destruction of the dilated and malformed capillary networks containing abundant photosensitizers at the lesion site without damaging the top normal epidermis containing little photosensitizers, the underneath dermal tissues are also protected because the limited laser penetration.

Photosensitizer is one of the key factors of PDT efficacy. Despite the significant results achieved with clinical application of the first generation of photosensitizers (hematoporphyrin derivatives, HpD), the skin phototoxicity reaction still lasts for 1~2 months and up to 3~4 months in some patients because HpD is a mixture of multiple porphyrins with strong affinity with collagen fibers,

its composition and chemical structure are yet to be clarified fully, and its excretion from the body is fairly slow. The patients must be strictly protected from light during this period, which severely affect not only the patient's quality of life and work but also cause great inconvenience in clinical treatment. Therefore, the current issue to be addressed urgently in order to meet the clinical demands on PDT is to develop photosensitizers with single chemical composition, clear structure, stable physicochemical properties, ideal action spectrum, high target tissue selectivity, strong photodynamic effect, low toxicity, and rapid metabolism.

Hemoporfin (hematoporphyrin monomethyl ether, HMME or MHD in short) co-developed by Shanghai Fudan-Zhangjiang Bio-Pharmaceutical Co., Ltd. and Chinese PLA General Hospital is a new drug for monomeric porphyrin photodynamic therapy. HMME methanol solution has characteristic absorption peaks at 401, 500, 533, 569 and 613 nm. HMME can be cleared from the tissues rapidly with very low phototoxicity in normal tissues. Both the acute toxicity and long-term toxicity of this compound are lower than the first-generation photosensitizers hematoporphyrin derivatives. The clear structure and rapid metabolism lead to mild adverse reactions, short period of protection from light, and treatments might be repeated within short intervals.

Based on the requirements of the drug clinical study approval notice number 2005L01082 issued by State Food and Drug Administration (SFDA), hemoporfin for injection phase III clinical trial is led by Peking University First Hospital and participated by Institute of Dermatology of Chinese Academy of Medical Sciences, The Ninth People’s Hospital of Shanghai Jiaotong University School of Medicine, Guangzhou General Hospital of Guangzhou Military Region, Huashan Hospital of Fudan University, Xijing Hospital of the Fourth Military Medical University, Union Hospital of Tongji Medical College, Huazhong University of Science and Technology, and Xiangya Hospital of Central South University. The study purpose is to evaluate the efficacy and safety of hemoporfin for injection co-developed by Shanghai Fudan-Zhangjiang Bio-Pharmaceutical Co., Ltd. and Chinese PLA General Hospital for the treatment of port-wine stains using PDT method.

Based on the phase II clinical trial results showing that 5mg/kg hemoporfin and photodynamic irradiation for 20min were safe and effective in the treatment of port-wine stains, the phase III clinical study is to further verify the efficacy and safety of PDT using hemoporfin for port-wine stains.

## 2. Trial purpose

This study is to evaluate the efficacy and safety of hemoporfin for injection for the treatment of port-wine stains using placebo as the control in two stages of PDT treatments. Primary endpoint will be established at the first stage, and safety analysis will include both stages.

## 3. Trial design

3.1 Design principle:

This is a parallel, placebo-controlled, double-blinded and multicenter clinical trial.

The subjects are randomized into trial group and control group at 3:1 ratio in this phase III clinical trial.

Statistical estimation of the sample size: using two-sided test of the efficacy, α=0.05 and β=0.1 (power=90%) based on phase II clinical trial results, the expert panel estimates the total clinical efficacy rate of hemoporfin for injection to be 75.0% in the treatment of port-wine stains and expects a 40% difference from the placebo group; consequently, the estimated minimal number of subjects is 66 for the trial group and 22 for the control group.

In compliance with the SFDA requirement of minimum sample size of no less than 300 subjects for phase III clinical trial group, and also taking into account of dropout, the sample size of this clinical trial was determined to be 330 subjects for the trial group and 110 subjects for the control group, giving a total of 440 subjects.

The subjects will be assigned into trial group or control group by stratified block randomization to receive not more than 2 courses of treatment. The control group receive placebo during the first course of treatment followed by trial drug during the second course of treatment. Each course of treatment will last for 8 weeks. Based on the phase II clinical trial results, the trial drug dose is set at 5mg/kg.

The trial design profile is shown in the following table.

| Trial period | Hemoporfin for injection dose | Group and number of cases | Design principle |
|---|---|---|---|
| The first course of treatment | 5mg/kg | 330 subjects in trial group | Randomized, double-blinded, placebo and parallel-controlled (multicenter) |
| | 0mg/kg | 110 subjects in control group | |
| The Second course of treatment | 5mg/kg | 330 subjects in trial group | Randomized, double-blinded (multicenter) |
| | 5mg/kg | 110 subjects in control group | |

3.2 Indication: port-wine stains

3.3 Randomization

In this randomized and double-blinded trial, each subject should be assigned a drug serial number strictly in accordance with the inclusion time sequence to determine the treatment group. Stratified block randomization was performed; and a software analysis system is used to generate continuous serial numbers corresponding to the sample size. The randomization numbers for each study center are generated at the same time. The sealed randomization table is maintained by the leading

institution and sponsor separately.

3.4 Trial plan: this clinical study is scheduled to complete between September 2008 and December 2009.

3.5 Subject selection

3.5.1 Inclusion criteria

All the patients participating in this clinical study must satisfy all the following criteria:

- The patients must been clinically diagnosed as port-wine stains;
- Male or female patients aged 14~65 years;
- The patients have read the subject's instructions and are willing to receive the treatment per protocol requirements and complete the visits in timely manner;
- The patients agree to sign the written informed consent form (ICF);
    - For subjects aged<18 years, both the subjects and legal guardians should sign the ICF at the same time.

3.5.2 Exclusion criteria

The patients who meet any one of the following criteria must be excluded from this clinical study:

- The affected area has other concomitant skin diseases (e.g. severe acne, contact dermatitis, or purulent infection) that may affect the efficacy evaluation;
- The treatment area has been treated with isotope therapy, PDT or other therapies that may affect the efficacy evaluation;
- The patients are currently suffering from allergic diseases; the patients have known photosensitive dermatitis and porphyria or known history of allergies to the trial drug (porphyrins) and drugs with similar chemical structure;
- The patients have allergic constitutions, scarring constitutions or scarring tendency;
- The patients used known photosensitive drugs such as griseofulvin, thiazide diuretics, sulfonylureas, phenothiazines, sulfonamides, retinoic acids, quinolones and tetracyclines within 1 month
- The patients have known severe immunocompromised conditions or require long-term use of corticosteroids and immunosuppressants;
- The patients have ECG abnormalities and organic heart diseases;

- Abnormal liver function (ALT and AST exceed the upper limit of normal (ULN), TBIL exceeds 1.5xULN) or severe renal dysfunction (renal function index exceeds 1.5xULN);
- The patients have hereditary or acquired coagulation disorders or are using anticoagulants;
- The patients have severe neurological, mental and endocrine disorders;
- The patients received systemic therapies to treat port-wine stains within 4 weeks prior to this treatment;
- The patients received topical therapies to treat port-wine stains within 2 weeks prior to this treatment;
- Pregnant and lactating women or women using inappropriate contraceptive measures;
- The patients participated in other drug clinical trials within 4 weeks prior to this treatment.

4. Trial materials and methods

4.1 Trial drug: hemoporfin lyophilized powder for injection, 100mg/vial, is manufactured by Shanghai Fudan-Zhangjiang Bio-Pharmaceutical Co., Ltd. There are totally 2 batches of trial drugs, the first batch number is 080601, manufacture date: June 2008, date of expiry: May 2010; the second batch number is 081201, manufacture date: Dec. 2008, date of expiry: Nov. 2010.

4.2. Trial light source: laser apparatus with a wavelength of 532nm, continuous or quasi-continuous output and maximum output power of no less than 5W.

4.3 Treatment methods

1) Hemoporfin solution is prepared per the trial dose. The prepared drug solution is a dark red transparent liquid and should be inspected for precipitation or discoloration phenomenon prior to injection.
2) The injection site skin is sterilized routinely.
3) Intravenous infusion of the drug solution should be at a constant speed using an infusion pump, and be completed within 20min. The relatively large veins easily protected from exposure to light should be selected (the largest vein in the arm such as the median cubital vein is preferred and small veins in the back of the hand should be avoided); 1ml physiological saline is injected for confirmation prior to drug infusion to prevent drug leakage into the extravascular space. Additional 2~4ml physiological saline should be infused at ending of drug infusion to decrease the intravascular drug concentration in order to prevent

photosensitive reactions at the injection site. The photosensitizers should be protected from light during the preparation and injection process to avoid efficacy decline. Measures should be taken to protect the subjects from light after drug infusion.

4) The target lesions are irradiated with 532nm laser and irradiation power density of 80-100 mW/cm$^2$.

   ☞ Irradiation time: the laser irradiation begins 10min after beginning of drug infusion and ends 20min later. The total irradiation time is 20 mintues.

   ☞ Precautions during laser irradiation: ① depending on the lesion size and site, the selected treatment areas should be as flat as possible; the hair-covered areas (eyebrow, beard and hairline) need to be shaved clean to avoid affecting laser irradiation. The non-treatment area surrounding the lesion, especially the nasolabial fold and other weak areas should be carefully covered with adhesive tapes and double-layer red-black clothing; ② the output power at the fiber terminal should be adjusted and measured according to the therapeutic regimen prior to irradiation, the beam expander should be maintained perpendicular to the irradiation surface; in addition, it is necessary to pay attention to the size of the beam spot, closely observe the changes in the irradiated areas, and accurately control the irradiation time; ③ the output power need to be monitored before, after and during the irradiation process.

5) Precautions: the patients should be instructed to observe the photoreaction after drug infusion and pay attention to photo protection. The photoreaction is expected to disappear completely in approximately 10 days after drug infusion. During the light protection period (2 weeks), the subjects should pay attention to protection of the exposed skin and eyes, avoid irradiation of sunlight or strong indoor lights. In case of drug extravasation during the infusion, the extravasation area must be kept completely away from light for a fairly long time until local swelling and discoloration disappears completely, otherwise severe local skin photoinjury may occur.

6) PDT may cause the following treatment reactions: the patients may experience itching, burning sensation, pain, swelling, blisters and scabs at the irradiated and surrounding area. These therapeutic responses are usually transient and self-healing without special treatment.

7) Adverse reactions: If light protection measures are not taken properly, some patients may experience photosensitive reactions such as photosensitive dermatitis, photosensitive ophthalmia, photosensitive cheilitis, etc; some patients may experience nausea, dizziness, headache, drowsiness, total bilirubin elevation, hyperpigmentation, hypopigmentation,

treatment site infection, scarring and other adverse reactions.

8) At week 8 after the first treatment, if the color of treatment area has not subsided completely and there are no serious pigmentation, scarring and other conditions that are unsuitable for re-treatment according to the investigator's judgment, the next stage treatment can be carried out.

4.4 Drug packaging and blinding

Because the drug used in this trial is powder for injection and the doses for the control group and trial group are different, stimulant is inappropriate for the double-blinded design; considering the fact that the trial drugs are prepared by nurses, the sponsor will prepare the drugs for each group per the double-blinded requirement during the drug preparation period.

Identical drug outer packaging are used for both groups; each patient needs an independent large packaging that contains two medium packaging marked with "drugs for the first course of treatment or drugs for the second course of treatment". Each medium packaging includes drugs for trial group (5 bottles of drugs in each medium packaging) and control group (each medium packaging contains 5 empty bottles for the first course of treatment and 5 bottles of drugs for the second course of treatment).

The statisticians will provide the random number table generated using DAS software simulation and provide it for random blinding of the drugs. Stratified block randomization is carried out by trial centers. Drug coding is completed by personnel unrelated to this clinical trial.

During the trial period, each center assigns a special person to manage the trial drugs and a nurses will prepare the trial drugs, keeping the investigators and subjects blinded.

4.5 Trial drug distribution and dispensing

Both the trial drug and light source are provided by Shanghai Fudan-Zhangjiang Bio-Pharmaceutical Co., Ltd. and delivered to all study centers periodically per the protocol by the company’s clinical study monitors. The drugs are distributed by a special person at each study center. The observation doctors should dispense the drugs by the order of each patient's visit and the sequence of drug numbers without choosing the drugs intentionally. The drug numbers remain unchanged throughout the trial process. After successful enrollment, the patients will be offered with sufficient dose of trial drug of identical labels.

The drug managers should fill out the drug dispensing/recovery registration form timely and accurately.

An emergency letter should accompany each of the trial drugs and the letters will be maintained by the principal investigator of the participating institution.

4.6 Drug counting

The investigators should accurately record the dose of each medication (bottle).

4.7 Drug storage

The study drug should be stored under 2~8℃ away from light in a special refrigerator, which should be locked and maintained by a special person. The investigators should return all the unused drugs to the monitors of Shanghai Fudan-Zhangjiang Bio-Pharmaceutical Co., Ltd. at ending of this trial.

4.8 Concomitant drugs

The subjects are not allowed use the drugs listed in the exclusion criteria during this trial. If concomitant drugs is indeed needed to treat other diseases, the name, dosage, indication, the beginning and ending date, and other relevant information should be recorded in the case report form (CRF).

5. The total number of subjects, grouping, randomization method and the tasks undertaken by each center

The total number of subjects planned to include is 440 and the distribution of these subjects among the centers are listed in the following table:

| Centre No. / Clinical study institution | Trail group | Control group | Total |
|---|---|---|---|
| 1. Peking University First Hospital | 39 | 13 | 52 |
| 2. Institute of Dermatology, Chinese Academy of Medical Sciences | 39 | 13 | 52 |
| 3. The Ninth People’s Hospital of Shanghai Jiaotong University School of Medicine | 57 | 19 | 76 |
| 4. Guangzhou General Hospital of Guangzhou Military Region | 39 | 13 | 52 |
| 5. Huashan Hospital of Fudan University | 39 | 13 | 52 |
| 6. Xijing Hospital of the Fourth Military Medical University | 39 | 13 | 52 |
| 7. Union Hospital of Tongji Medical College, Huazhong University of Science and Technology | 39 | 13 | 52 |
| 8. Xiangya Hospital of Central South University | 39 | 13 | 52 |
| Total | 330 | 110 | 440 |

The number of subjects can be adjusted according to the inclusion rate at each center.

6. Trial arrangement

- Treatments: up to 2 times (the patients can receive an additional treatment if the treatment area did not subside completely after the first treatment)
- Efficacy evaluation: the efficacy evaluation is carried out in Week 8 after each treatment; both

evaluations are compared to the disease conditions before the treatment.

- Laboratory examination: the testing is carried out before each treatment and on Day 4 after the treatment.

Trial Flowchart

| | PDT treatment | | | | Efficacy observation |
|---|---|---|---|---|---|
| | The first course of treatment | | The Second course of treatment | | The first/second course of treatment<br>Week 8 of treatment |
| | Day 1 | Day 4 | Day 1 | Day 4 | |
| Instructions for patients | × | | | | |
| Signing ICF | × | | | | |
| Laboratory examination | × | × | × | × | |
| Inclusion criteria | × | | | | |
| Exclusion criteria | × | | | | |
| History | × | | | | |
| Accompanied diseases | × | | | | |
| Therapies | × | | | | |
| PWS type | × | | | | |
| Photography of the treatment area | × | × | × | × | × |
| Allocation of drug numbers | × | | | | |
| PDT treatment | × | | × | | |
| Observation of photoreaction | × | × | × | × | × |
| Observation of adverse event | × | × | × | × | × |
| Evaluation of efficacy | | | × | | × |
| Investigator's evaluation | | | × | | × |
| Subject's evaluation | | | × | | × |
| Changes of concomitant therapies | | × | × | × | × |

7. Observation of the subjects

- Observation and evaluation of the same subject should be carried out and recorded carefully by the same investigator as far as possible.

- The treatment area of all patients should be photographed before and after the treatment at each visit. The sponsor will provide uniform labels to mark the subject's number, date, and number of treatments on the photograph.

7.1 The patient's general conditions (including the initials, date of birth, body height, body weight, vital signs, etc.), other comorbidities and history of treatment, and the onset history of port-wine stains (including past medical history, course of disease, affected sites, areas, treatments, etc.) are recorded at enrollment.

7.2 typing type of port-wine stains in the lesion area should be assessed at enrollment

Based on the erythema color and skin hyperplasia, port-wine stains can be divided into three types:

- Red type: the lesion is flat, not raised, similar to normal skin, the color is light pink to dark red and fades completely on finger pressing.
- Purple type: the lesion is flat, not raised, similar to normal skin, the lesion is pale to dark purple and the color can fade completely or incompletely.
- Hypertrophictype: the lesion is thickening and raised above normal skin with nodular hyperplasia, the color is dark purple-red and fades completely or incompletely.

7.3 Laboratory examination: all subjects should receive the following examinations:

- Routine blood tests: Included WBC, RBC, HGB, PLT.
- Routine urine tests: including urine proteins and urine glucose, WBC, RBC.
- Blood biochemical tests: Included ALT, AST, BUN, CR, TBiL.
- ECG recording:
- Urine pregnancy test (only for female patients of childbearing age without using contraceptive measures)

7.4 Observation of concomitant drug: record the name, indication, dosage, route of administration, start and end date of the concomitant drugs used by patients during their participation in this clinical study.

8. Efficacy evaluation

8.1 Primary efficacy

The expert panel will determine the efficacy based on the photos taken before and after treatment per the following criteria.

- Almost cured: color of the treated area subsided basically (the level of improvement≥90%);
- Significantly improvement; color of the treated area subsided obviously (the level of improvement≥60%, <90%);
- Some improvement: color of the treated area subsided partially; (the level of improvement≥20%, <60%);
- No improvement: color of the treated area did not change essentially. (the level of improvement<20%);

8.2 The investigators and patients will subjectively evaluate the efficacy as excellent, good, moderate and insufficient and safety as excellent, good, moderate and unsatisfied, separately.

9. Safety

9.1 The adverse events (AE) are identified through inquiries of the subjects (caution: avoid inductive questioning, phrases such as "how do you feel after the treatment?" can be used during the inquiry), physical examination and comparisons of the laboratory examination results before and after the treatment including blood routine, urine routine, liver function, kidney function, ECG recording.

9.2 Adverse events

9.2.1 Definition: “AE” is any adverse medical events occurred after the subjects have received the drug, which may be unrelated to the treatment. Accordingly, AE can be any discomforts and unaware signs (e.g. abnormal laboratory examination results), symptoms or diseases.

- ☞ All AEs must be recorded in CRF regardless of their severity and relationship with the used drug. For all the subjects who withdraw from this study due to AEs, the investigators should continue to follow-up with them until full recovery, record the relevant data, and indicate AE occurrence date and recovery date in the CRF.

9.2.2 Correlation assessment: the investigators should evaluate the correlation of the AEs with drug treatment in this trial as follows:

✧ Certainly related to the trial drug

Evidence of using the trial drug; plausible time relationship between the occurrence of AE and the use of trial drug; the occurrence of AE can be more reasonably explained by the use of trial drug than other reasons; plausible response to withdrawal; positive drug rechallenge test; consistence between the AE pattern and the past understanding of this drug or this type of drugs.

✧ Probably / Likely related to the trial drug

Evidence of using the trial drug; plausible time relationship between the occurrence of AE and the use of trial drug; the occurrence of AE can be more reasonably explained by the use of trial drug than other reasons; reasonable drug withdrawal response;

✧ Possibly related to the trial drug

Evidence of using the trial drug; plausible time relationship between the occurrence of AE and the use of trial drug; AE may be explained by the use of trial drug or other reasons; reasonable drug withdrawal response.

✧ Unlikely related to the trial drug

Evidence of using the trial drug; AE is more likely caused by other reasons; drug withdrawal reaction is negative or plausible; drug rechallenge test is negative or plausible.

✧ Not related to the trial drug

The subject did not use the trial drug; the time relationship between the occurrence of AE and the use of trial drug is not plausible; or some other obvious reason causes the AE.

9.2.3 Severity evaluation

The intensity or severity of AE falls into the following categories:

- ✧ Mild: easily tolerated AE, subjects can feel it occasionally.
- ✧ Moderate: AE is significantly discomfort and interfere with the daily activities; the subjects felt significantly but still can tolerate the symptoms without need to discontinue drug treatment.
- ✧ Severe: AE significantly interferes with normal daily activities; the subjects felt significant intolerable symptoms and discontinuation of drug treatment is required.

9.2.4 AE report

- ✧ The investigators need to fill out the AE record form in CRF.
- ✧ The data retained in CRF will be forwarded to the clinical trial monitors by the end of this trial.

9.2.5 Handling of AE during and after the trial period

The investigators should observe and record the outcomes of all AEs and follow up with the subjects who withdraw from this trial due to AE until the AE is resolved completely. The investigators must determine whether the AE is related to the study drug and provide evidence to support the opinion.

9.3 Severe adverse events (SAE) and unexpected adverse drug reactions (UADR)

9.3.1 Definition: SAE is one of the adverse events below and might occur at any doses:

- ✧ Causing death
- ✧ Life-threatening
- ✧ Requiring hospitalization or prolonged stay in hospital
- ✧ Causing long-term or significant disability, or affecting working and living capabilities
- ✧ Causing congenital malformations

“UADR” refers to an adverse reaction, the nature or severity of which is not consistent with the applicable product information.

9.3.2 SAE reporting

- ✧ All SAEs must be notified by phone or fax within 24 hours to the principal investigator: Zhu Xue-jun [Tel: (010) 66551216，Fax: (010) 66551216], Department of Dermatology and Venereology, Peking University First Hospital and the supervisor of this clinical study sponsor: Tao Ji-ning[Tel: (021) 58553593, 13641733664，Fax: (021) 58553990], and reported to SFDA and local Food and Drug Administration of the provinces, autonomous regions and municipalities.
- ✧ The complete SAE Report Form should be filled within 3 days and submitted to the sponsor's clinical study monitors, the principal investigator and the Medical Ethics Committee of Peking University First Hospital for record-filing purpose.
- ✧ If the AE is severe and life threatening, the written event summary should be submitted to the relevant regulatory authority within 7 days; if the AE is severe but not life threatening, the summary can be submitted within 15 days.

9.3.3 Opening the emergency letters and unblinding:

Blinding should be well kept throughout the study. However, in case of emergency and when it becomes really necessary to identify the type of drug a patient has received, the principal investigator of the study institution can open the emergency letter (in each set of drugs) which contains the drug name and number for this patient. The monitors should be notified of the process immediately. The investigators should record in CRF the reason and date of unblinding in detail and sign on the record. Once unblinded, the patient will have to be discontinued in this trial and treated as a dropout case.

9.3.4 The observer should provide the following information in SAE reports:

- ✧ Observer's name and center code
- ✧ Initials of the Subject's name
- ✧ Subject's general data including age, date of birth, marital status and body weight
- ✧ SAE details including descriptions of the affected body site, severity and the criteria for determining severity
- ✧ Description
- ✧ Occur date

- ✧ End date or duration of the AE
- ✧ Severity
- ✧ Treatment (including hospitalization)
- ✧ Causal relationship with the trial drug
- ✧ Outcome: recover or having sequelae
- ✧ Adjustment of drug treatment
- ✧ Measures taken for trial drug such as stopping treatment or continuing treatment, etc
- ✧ For life-threatening events, the cause of death and the possible relationship should be provided
- ✧ Autopsy findings
- ✧ Drug number
- ✧ Indication of the used drug or the trial drug
- ✧ Formula or concentration of the drug
- ✧ Daily dose and regimen
- ✧ Route of administration
- ✧ Other treatments: the investigators should provide information on the relevant products for concomitant drugs (including over-the-counter drugs) and non-drug therapies
- ✧ Other information: any information that can help understanding the events such as allergy, drug or alcohol dependence, family history, special examination findings.

10. Subject's withdrawal

10.1 Determination of withdrawal: any patient who has filled in the ICF and been enrolled into the clinical trial after screening has right to withdraw from this trial at any time; the subject will be regarded as withdrawal case as long as they has not completed the endpoint observation of this trial regardless of the withdrawal time and reasons.

10.2 Common causes for subject's withdrawal

10.2.1 Withdrawal decided by the investigators

When it is no longer appropriate for the subjects to continue trial as a result of trial-emerged situations, the investigators can decide the withdrawal of the subject from this trial. The common reasons are as follows:

- ✧ A subject discrepant with the inclusion criteria were included by mistake
- ✧ Allergic reactions or disease deterioration which need emergency measures
- ✧ Poor compliance of a subject, who fails to use the study drug per requirements, and violate the trial protocol
- ✧ In the investigator's opinion, continuing the treatment is not to the patient's best interests (e.g. pregnancy)
- ✧ The efficacy can't be evaluated or the efficacy or safety evaluation is affected by reasons like data incompleteness, etc
- ✧ Unblinding or emergency unblinding
- ✧ It is inappropriate for the subjects to continue this trial due to AE or SAE

10.2.2 Withdrawal decided by subjects

The subjects have rights to withdraw during this trial, or although the subjects did not explicitly request to withdraw from this trial, they will be regarded as "withdrawal" (or "dropout") cases if they no longer take the study drug or examination and consequently lost to follow-up. Efforts should be made to find out and record the reasons of withdrawal such as subjective perception of poor efficacy; finding it difficult to tolerate certain adverse reactions; too busy to continue this clinical study; economic reasons; or lost to follow-up for unexplained reasons.

10.3 Handling of the withdrawal cases

The investigators should fill in the CRF with the withdrawal reasons, try best to contact the patients to complete the assessment items, fill in the last visit form, and record the last dosing time. For withdrawals due to AEs that have been eventually judged to be related to the trial drug through follow-ups, the investigators must record them in the CRF and notify the sponsor. CRF of the withdrawal cases must be retained regardless of their withdrawal reasons; the last assessment results will be carried forward to serve as the final results for full data analysis of the efficacy and adverse reactions.

11. Discontinuation of the trial

Trial discontinuation refers to stop of this whole clinical trial without completion per the trial protocol. The major purposes of trial discontinuation are to protect the subject's interests, ensure trial quality and avoid unnecessary financial loss. Trial discontinuation during the study can result from the following reasons:

- ☞ The investigators identify serious safety issues

- ☞ Very poor efficacy makes it unnecessary to continue this trial
- ☞ There are major defects in protocol design or major deviations during the implementation process
- ☞ The sponsor has budget or management difficulties
- ☞ The state's administrative department revokes this trial

The trial discontinuation can be temporary or permanent. When the trial is discontinued, all the trial records should be retained for future supervision.

## 12. Clinical trial quality control

During this study, all the clinical trial participants should implement the GCP principles and strictly follow the clinical protocol in observation and evaluation. The sponsor will dispatch clinical study monitors to inspect the trial progresses and CRF quality and give advice on the drugs and study-emerged issues.

- ➢ The participating investigators must receive uniform training and use uniform recording style and determination criteria.
- ➢ The entire clinical trial process should proceed under strict blinded condition.
- ➢ The investigators should record the contents of all raw data items accurately, carefully and in sufficient details, and fill in the CRF items accurately.
- ➢ All the observation results and findings in this clinical trial should be verified to guarantee the data reliability and ensure that all the conclusions of this clinical trial are derived from the raw data. Corresponding data management procedures should be in place in all stage of clinical trial and data processing.
- ➢ Active measures should be taken in case of potential dropout cases. The dropout rate should be controlled within 10%.
- ➢ All the study hospitals should maintain their quality control standards stringently, and the clinical monitors will carry out periodical monitoring.

## 13. Data management

See “data management plan” for details of data management. Before database lock, the data manager, principal investigator and sponsor will carry out blind review and discussion, and come into a final data management report. This protocol will only provide the general requirements for data management.

### 13.1 The filling and transfer of CRF

The completed CRF will be reviewed by the clinical investigators and monitors before transferring to the data statistical processing institution for data entry and management. All the processes should be documented properly.

13.2 Data entry and modification

The data managers of the statistical institution are responsible for data entry and management using excel or EpiData2.1 database. The data are required to be double entered by two independent data managers and verified to ensure data accuracy.

The data manager will fill out the DRQ in response to any doubts in the CRF and send queries to the investigators through clinical monitors; the investigators should answer the questions promptly and respond back. The data manager will modify the data per the investigator's response, verify and enter the modifications, and re-issue the DRQ if necessary.

13.3 Blind review and database lock

The principal investigator, sponsor and statistical analyst will lock the database after completing the blind review and confirming correctness of the constructed database.

13.4 Unblinding

Once all the study data have been reviewed and locked, the principal investigator (PI), statisticians and sponsor will jointly discuss the statistical plan, unblind this study and co-sign the blind codes.

Any modifications of the database after unblinding must be approved by the principal investigator, biostatisticians and data manager in written prior to implementation.

Once received the blind codes, the statistical analysts will carry out statistical analysis per the statistical plan and eventually write the statistical analysis report based on which the principal investigator will write the clinical trial summary report.

14. Statistical analysis

See the "statistical analysis plan" for details of statistical analysis. Before database lock, the statisticians, principal investigator and sponsor will jointly discuss and finalize the statistical plan in accordance with the data characteristics. This protocol only provides the general statistical requirements.

14.1 Primary Populations

The primary efficacy variable will be summarized and analyzed in ITT and PP populations, the secondary efficacy variables will be summarized and analyzed in ITT populations. ITT population will be the primary analysis population.

✧ Full analysis set (FAS): refers to the set of eligible cases and dropout cases excluding the

rejected cases. The ITT population will be defined as all randomised patients having received at least one dose of either the tested or the reference treatments.

- ✧ Per protocol set (PPS): The PP population will be defined as all the subjects who completed the treatment period without any major deviation from the protocol.
- ✧ Safety set (SS): Safety population will be defined as all the subjects who received at least one treatment and have actual data of safety indexes.

14.2 Statistical Analysis Method

14.2.1 Demographic Characteristics

(1) Consistency with normal distribution: Because this is a randomized and blinded trial with a large sample size, the data is acceptable when close to normal distribution. Appropriate statistical methods or data transformation will be adopted for non-normal distributed data, D method, W method or moment method will be used.

(2) Presence of outliers: statistical and clinical analysis should be carried out to determine the acceptation and rejection.

(3) Handling of the missing values of primary efficacy index: if the primary efficacy data of an individual subjects is missing, missing data were imputed as non-responses.

(4) Analysis of the subjects who did not complete this trial: the cause of each dropout case should be analyzed.

(5) Descriptive statistics: provide the mean, standard deviation, maximum, minimum, median, confidence interval, frequency (constituent ratio), etc.

14.2.2 Statistical inference method

(1) Measurement data: t-test, paired t-test, rank sum test, paired rank sum test, median test method, etc.

(2) Numeration data: chi-square test, Fisher's exact test, etc.; Ridit analysis and CMH method can be used to test the ranked data.

(3) Primary efficacy: The response rate will be analyzed using Cochran–Mantel–Haenszel test. Logistic regression analysis, with center and PWS type as covariates will be used to compare treatment group versus placebo group on primary efficacy. If baseline variables are not balanced between groups, non-balanced variable will be also included as covariates in the model. For response rate at week 8, missing data were imputed as non-responses.

(4) Difference analysis: in primary efficacy analysis, a 40% difference between the treatment and placebo group in response rate is expected to be detected.

14.2.3 Statistical presentation

(1) The report mainly uses the self-evident tables that include title, notes and number of cases.

(2) The results of repetitively measured data use both tables and the annexed statistical charts at the same time to improve readability.

(3) Difference analysis is represented by the two-sided 95% confidence interval for the intergroup difference in efficacy; all the general statistical tests are two-sided tests, $P \leq 0.05$ is considered to have statistical significance.

14.3 Statistical software

All statistical analyses will be carried out by the SAS statistical software package (version 9.1.3).

14.4 Interim analysis

This study does not have interim analysis. If special circumstances including uncertain efficacy or safety issues emerge during this trial, the sponsor, investigators and statisticians will jointly discuss the issues and make decisions.

14.5 Contents of statistical analysis

1. Case distribution: provide the sizes of different data sets of all groups, distribution of the cases among all centers, comparison of the total dropout rate, and a detailed listing of the reasons for termination.

2. Comparability analysis: demographic data and other baseline indexes are compared to measure the intergroup comparability.

3. Compliance analysis: (1) Drug compliance analysis: compare whether the patients of the two groups have taken the correct doses of trial drugs in timely manner without using the prohibited drugs and food as described in this protocol. (2) Drug combination analysis: count the number of subjects receiving drug combination in each group and provide a detailed list.

4. Efficacy analysis: PP and ITT analysis of the primary efficacy should be carried out at the same time; because this study is a multicenter clinical trial, the impact of center effect on the efficacy should be considered during analysis.

5. Analysis of the efficacy influencing factors: if there are significant differences in age, gender, disease types, disease conditions and other factors between the two groups before drug treatment or relevant factors significantly affecting the efficacy (e.g. drug combination) during the trial process, these factors should be used as covariates for analysis of covariance or logistic regression analysis during intergroup efficacy comparison.

6. Safety analysis: based on the requirements for correlation of adverse reactions, use listings to describe the AEs and adverse reactions in both groups (including the number of cases of various

AEs, the number of cases with "normal shift to abnormal" or "exacerbation of the abnormality" of the laboratory test indexes and abnormal conversion rate before and after this trial), list the causes and explain. Chi-square test is used for statistical analysis of the adverse reactions.

## 15. Ethics

### 15.1 Ethics committee

The trial protocol and ICF must be approved by the “Medical Ethics Committee of Peking University First Hospital”; the approval notice issued by the ethics committee must be obtained before initiating this trial.

### 15.2 GCP guideline

Implementation of this trial complies with the national GCP guidance to protect the subject's rights, safety and interests effectively and ensure the reliability of clinical trial data.

### 15.3 Subject’s ICF

The investigators must explain all the ICF contents approved by the ethics committee to the subjects before initiating this trial and obtain the written informed consent signed and dated by the subjects or their legal representatives; a copy of the ICF must be made available to the subjects after obtaining the informed consent.

If both the subjects and their legal representatives are illiterate, a witness should be present throughout the informed process. After a detailed explanation of the ICF, the subjects or their legal representatives can give verbal consent and the witnesses should sign and date the consent form. When it is impossible to obtain the subject's consent in advance in case of specialty (e.g. emergency), it is necessary to obtain consent from the legal representatives and inform the subjects or their legal representatives of the relevant trial situation as early as possible; the trial can proceed if they give their consent.

## 16. Data storage

For purpose of evaluation and supervision by the SFDA and sponsor, the investigators should save all the study data including all the patient’s ICF and CRFs, detailed drug dispensing records and other materials with confirmation by the participating subjects (for effective verification of different recorded data such as CRF and hospital's original records) and the original signatures. The investigators should maintain the above data for 5 years after the ending of this clinical trial.

The ownership of all data of this clinical trial belongs to the sponsor Shanghai Fudan-Zhangjiang Bio-Pharmaceutical Co., Ltd.; unless required by the SFDA, the investigators should not disclose

these data to any third party by any means without written approval of the sponsor. Approval by the sponsor in advance is required when the investigators publish the study results in scientific journals or present them at academic conferences. The sponsor has the rights to use these reports for academic promotion purposes.
