# Supplementary material for: Hemoporfin Photodynamic Therapy for Port-Wine Stain: A Randomized Controlled Trial
Source: PLoS One. 2016 May 26;11(5):e0156219. doi: 10.1371/journal.pone.0156219 (PMC4881994; doi:10.1371/journal.pone.0156219)
Supplement: S3 File — (DOCX) [file pone.0156219.s004.docx]

**S3 File. Standard operation procedure for digital photography used in this study.**

1. Preparation of the participant and the target treatment site:
   1. Clean the targeted site: remove cosmetics and accessory items(i.e. glasses, hat, neckerchief or jewelry), bind the hair with a fillet, if necessary;
   2. Mark the targeted site;
   3. Label the targeted site with the specially-designed inert color marker.
2. Prepare the environment for photography:

In a dark room with a ceiling-mounted fluorescent lamp, place two luminescence lamps (85W) at 45° to the left and right of the participant and at a distance of 1.5 m each from the participant.

1. Prepare the camera for image capture:
   1. Use a digital camera of no less than 5 mega pixels;
   2. Set the picture size at no less than 2272×1704;
   3. Use aperture-priority mode, and set the aperture to 8 or 11;
   4. Use the flash, and AVOID surface glare on the targeted site.
2. Image capture:
   1. Keep the target site at an equal level with both light sources;
   2. The camera should be fixed (by a tripod) at 1m away from the participant and at the same level as the target site;
   3. The targeted site should be at the center of the picture, and should occupy the majority of the picture area;
   4. Take pictures at 90° and then at 45°to the left and right of the treated surface;
   5. Take more than one picture at each point, and check the quality of the image acquired before moving on.
3. All conditions above were required to be consistent throughout the study.
4. All digital images should be transferred to the computer as quickly as possible and back-up should be made frequently.
5. Any modification of the original image was strictly prohibited.
