# Supplementary material for: Hemoporfin Photodynamic Therapy for Port-Wine Stain: A Randomized Controlled Trial
Source: PLoS One. 2016 May 26;11(5):e0156219. doi: 10.1371/journal.pone.0156219 (PMC4881994; doi:10.1371/journal.pone.0156219)
Supplement: S1 Table — (DOCX) [file pone.0156219.s005.docx]

- - - 1. S1 Table. Details of laser instruments used in this study

| **Study site** | **Manufacturer/Country** | **Model** | **Type/Mode** | **Power output** |
| --- | --- | --- | --- | --- |
| 1, 4, 7 | Wuhan Huagong Laser Engineering Co., Ltd./China | HGL-MYK8 | Nd: YAG, ^FD^ (KTP)/Continuous | 0~8W |
| 3 | LASERING S.R.L./Italy | VELURE S5 | Nd:YVO4, ^FD^ (LBO)/Continuous | 0.1~5W |
| 2, 5 | WaveLight Aesthetic GmbH/Germany | IDAS | Nd:YVO4, ^FD^ (LBO)/Continuous | 0.5~8W |
| 6,8 | Tongye Technologies Development Co., Ltd./China | TY532-150 | Nd: YAG, ^FD^ (KTP)/Continuous | 0.5~15W |

Abbreviation: FD, frequency doubled.

Denotations: 1: Peking University First Hospital, Beijing; 2: Chinese Academy of Medical Sciences and Peking Union Medical College, Nanjing; 3: Ninth People’s Hospital Affiliated to Shanghai Jiao Tong University School of Medicine, Shanghai; 4: The General Hospital of Guangzhou Military Command, Guangzhou; 5, Huashan Hospital, Fudan University, Shanghai; 6, Xijing Hospital, Fourth Military Medical University, Xi’an; 7, Union Hospital of Tongji Medical College of Huazhong University of Science and Technology, Wuhan; 8, Xiangya Hospital Hunan Medical University, Changsha.
