# Supplementary material for: Hemoporfin Photodynamic Therapy for Port-Wine Stain: A Randomized Controlled Trial
Source: PLoS One. 2016 May 26;11(5):e0156219. doi: 10.1371/journal.pone.0156219 (PMC4881994; doi:10.1371/journal.pone.0156219)
Supplement: S2 Table — (DOCX) [file pone.0156219.s006.docx]

- - - 1. S2 Table. Stratified analysis of efficacy at week 8 for the patients who received hemoporfin in stage 1

| **Stratification factors** | **Number of patients, *n*** | **Grading of PWS fading and response rates, *n* (%)** | | | | | |
| --- | --- | --- | --- | --- | --- | --- | --- |
|  |  | **NI**  **(<20%)** | **SI**  **(20-59%)** | **GI**  **(60-89%)** | **CR**  **(≥90%)** | **At least GI**  **(≥60%)** | **At least SI**  **(≥20%)** |
| Sex |  |  |  |  |  |  |  |
| Male | 138 | 20 (14.5) | 75 (54.3) | 36 (26.1) | 7 (5.1)^a^ | 43 (31.2)^b^ | 118 (85.5) |
| Female | 191 | 14 (7.3) | 77 (40.3) | 70 (36.6) | 30 (15.7)^a^ | 100 (52.4)^b^ | 177 (92.7) |
| Age in years |  |  |  |  |  |  |  |
| 14-18 | 48 | 6 (12.5) | 15 (31.3) | 20 (41.7) | 7 (14.6) | 27 (56.3) | 42 (87.5) |
| 19-30 | 220 | 21 (9.5) | 109 (49.5) | 66 (30.0) | 24 (10.9) | 90 (40.9) | 199 (90.5) |
| 31-65 | 61 | 7 (11.5) | 28 (45.9) | 20 (32.8) | 6 (9.8) | 26 (42.6) | 54 (88.5) |
| Location |  |  |  |  |  |  |  |
| Centrofacial | 162 | 19 (11.7) | 89 (54.9) | 40 (24.7) | 14 (8.6)^c^ | 54 (33.3)^d,f^ | 143 (88.3) |
| Non-centrofacial | 105 | 12 (11.4) | 44 (41.9) | 38 (36.2) | 11 (10.5) | 49 (46.7)^d,e^ | 93 (88.6) |
| Neck | 60 | 3 (5.0) | 17 (28.3) | 28 (46.7) | 12 (20.0)^c^ | 40 (66.7) ^e,f^ | 57 (95.0) |
| Other | 2 | 0 (0.0) | 2 (100) | 0 (0.0) | 0 (0.0) | 0 (0.0) | 2 (100.0) |
| Type |  |  |  |  |  |  |  |
| Pink | 111 | 12 (10.8) | 39 (35.1) | 40 (36.0) | 20 (18.0)^g,i^ | 60 (54.1)^h,j^ | 99 (89.2) |
| Purple | 189 | 15 (7.9) | 97 (51.3) | 60 (31.7) | 17 (9.0)^g^ | 77 (40.7)^h,k^ | 174 (92.1)^l^ |
| Hypertrophic | 29 | 7 (24.1) | 16 (55.2) | 6 (20.7) | 0 (0.0)^i^ | 6 (20.7)^j,k^ | 22 (75.9)^l^ |

Abbreviations: NI, no improvement; SI, some improvement; GI, great improvement; CR, nearly completely resolved.

Denotations: a-l, *P*<0.05 for comparisons between each of the two numbers with the same superscript.
