# Supplementary material for: Hemoporfin Photodynamic Therapy for Port-Wine Stain: A Randomized Controlled Trial
Source: PLoS One. 2016 May 26;11(5):e0156219. doi: 10.1371/journal.pone.0156219 (PMC4881994; doi:10.1371/journal.pone.0156219)
Supplement: S3 Table — (DOCX) [file pone.0156219.s007.docx]

- - - 1. S3 Table. Stratified analysis of efficacy based on PWS type at week 8

| Response | Type/severity | PDT-hemoporfin | | PDT-placebo | | P value^*^ |
| --- | --- | --- | --- | --- | --- | --- |
|  |  | n | % | n | % |  |
| at least SI |  |  |  |  |  |  |
|  | Pink | 111 | 89.2 | 49 | 26.5 | <0.001 |
|  | Purple | 189 | 92.1 | 56 | 25.0 | <0.001 |
|  | Hypertrophic | 29 | 75.9 | 5 | 0.0 | 0.003 |
| at least GI |  |  |  |  |  |  |
|  | Pink | 111 | 54.1 | 49 | 2.0 | <0.001 |
|  | Purple | 189 | 40.7 | 56 | 0.0 | <0.001 |
|  | Hypertrophic | 29 | 20.7 | 5 | 0.0 | 0.559 |
| CR |  |  |  |  |  |  |
|  | Pink | 111 | 18.0 | 49 | 0.0 | 0.001 |
|  | Purple | 189 | 9.0 | 56 | 0.0 | 0.030 |
|  | Hypertrophic | 29 | 0.0 | 5 | 0.0 | >0.999 |

Abbreviations: SI, some improvement; GI, great improvement; CR, nearly completely resolved.

Denotations: ^*^ All comparisons were conducted using chi-square or Fisher’s exact test.
